# Supplementary material for: Bioinspired design of Na-ion conduction channels in covalent organic frameworks for quasi-solid-state sodium batteries
Source: Nat Commun. 2023 May 27;14:3066. doi: 10.1038/s41467-023-38822-w (PMC10224921; doi:10.1038/s41467-023-38822-w)
Supplement: Supplementary file 1 — Supplementary Information [file 41467_2023_38822_MOESM1_ESM.pdf]

Supplementary Information for

Bioinspired design of Na-ion conduction in covalent organic frameworks for quasi-solid-state sodium batteries

Yingchun Yan[1], Zheng Liu[1]*, Ting Wan[1], Weining Li[1], Zhipeng Qiu[1], Chunlei Chi[1], Chao Huangfu[1], Guanwen Wang[1], Bin Qi[1], Youguo Yan[1]*, Tong Wei[1], and Zhuangjun Fan[1]*

[1]School of Material Science and Engineering, China University of Petroleum, Qingdao 266580, China.

E-mail: liuzhengbeyond@163.com, yyg@upc.edu.cn, fanzhj666@163.com

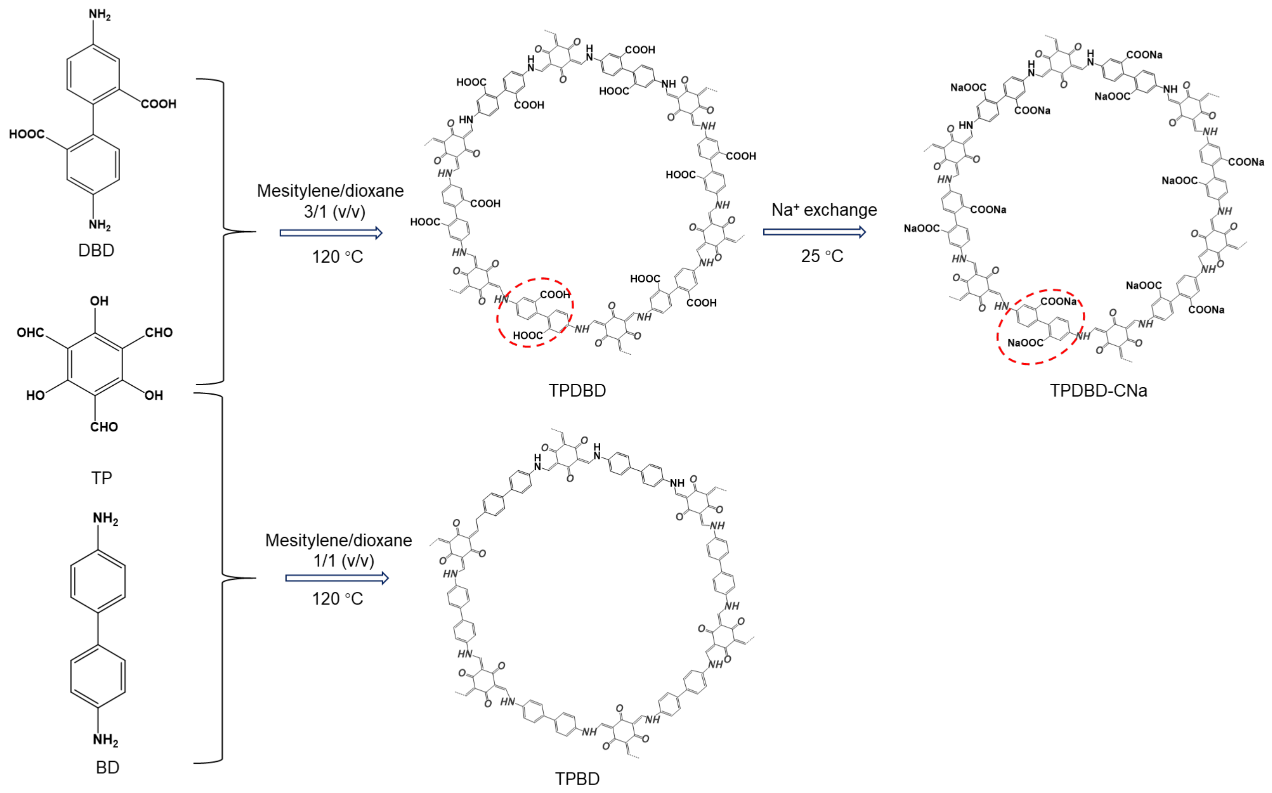

Supplementary Figure 1. Synthetic scheme of TPDBD, TPDBD-CNa, and TPBD.

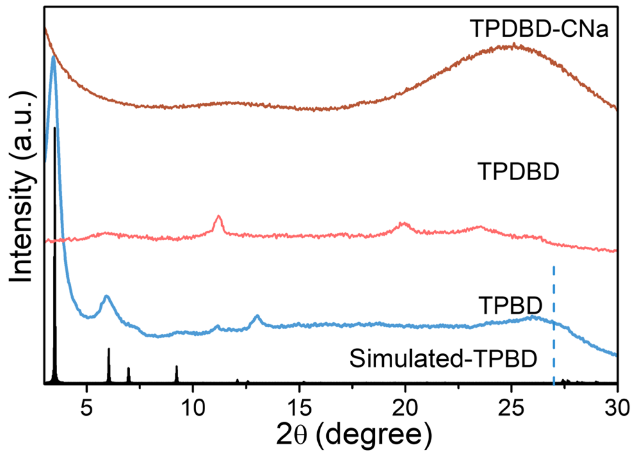

Supplementary Figure 2. PXRD patterns for TPBD, TPDBD, TPDBD-CNa, and simulated TPBD.

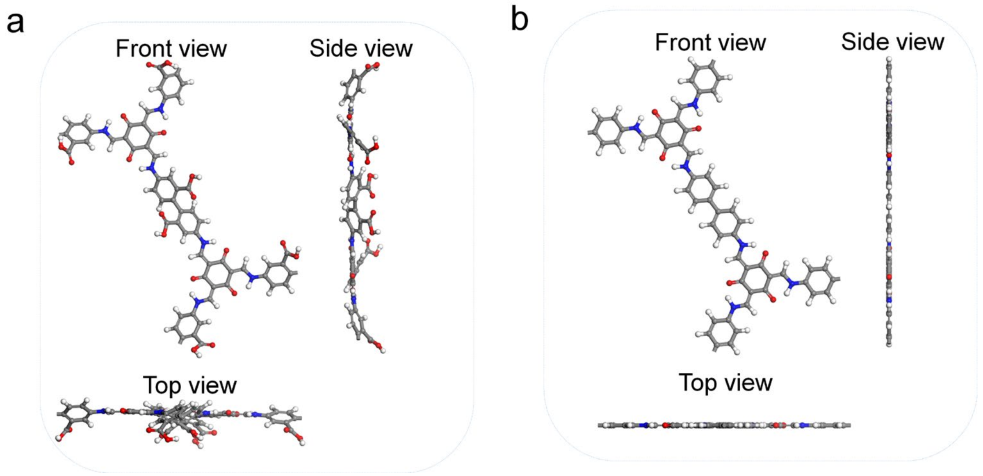

Supplementary Figure 3. Optimized structure of TPDBD (a) and TPBD (b) (the white, blue, gray and red spheres denote hydrogen, nitrogen, carbon, and oxygen, respectively).

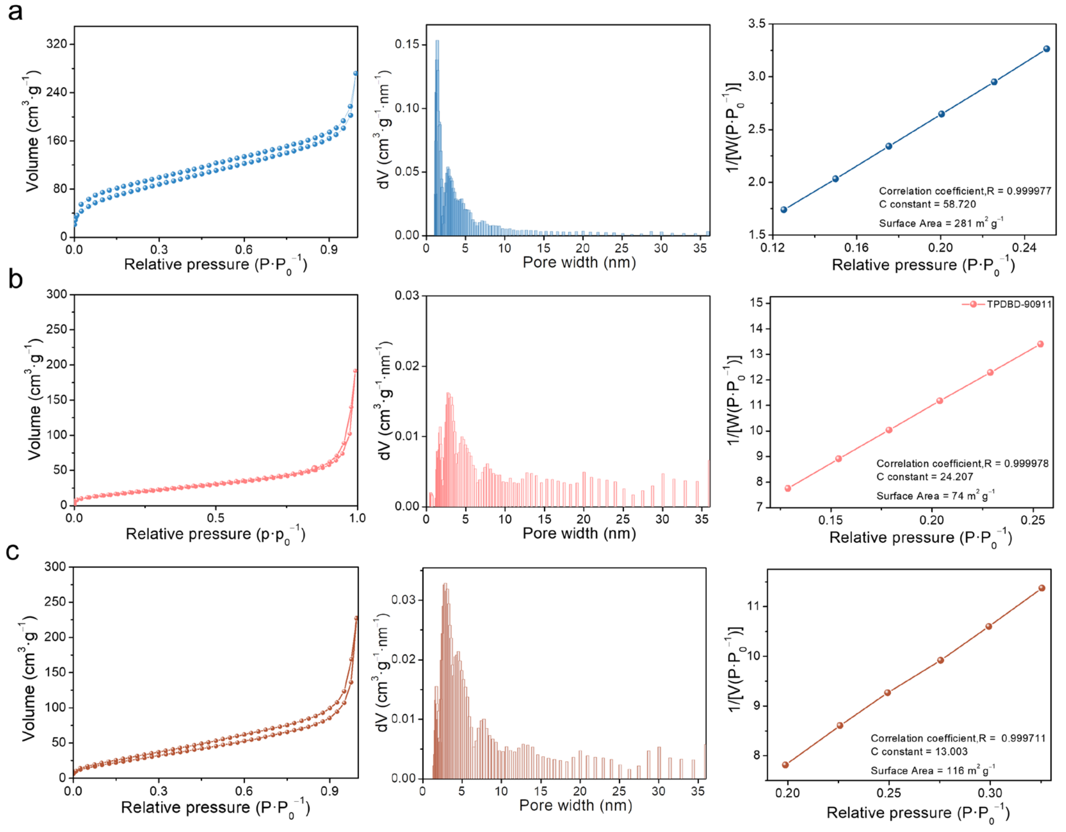

Supplementary Figure 4. N_2 adsorption and desorption curves and pore size distributions of TPBD (a), TPDBD (b), and TPDBD-CNa (c).

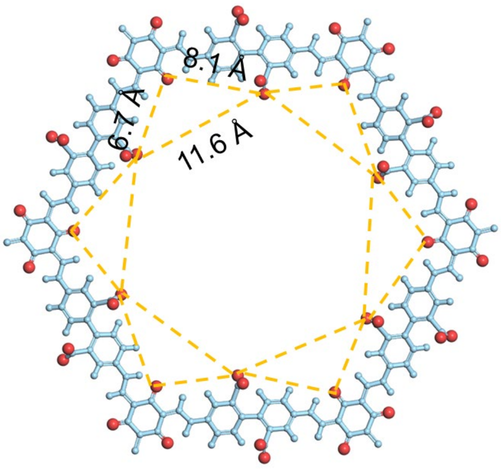

Supplementary Figure 5. Distances between −COO⁻ of optimized structure (the red spheres denote oxygen and cyan sticks denote covalent organic framework).

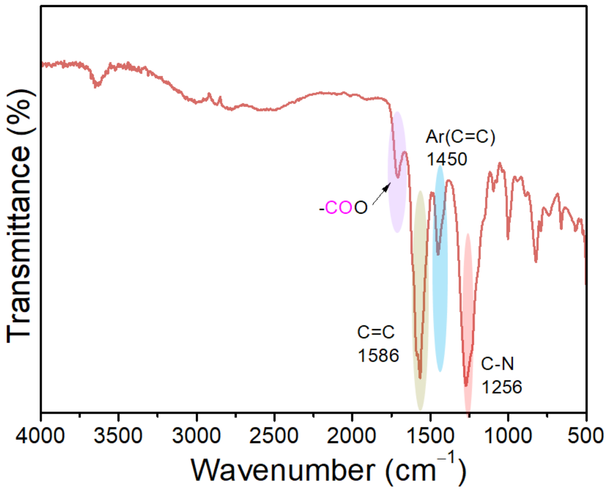

Supplementary Figure 6. FTIR spectrum of TPDBD-CNa measured at 25 ± 1 °C.

1 °C.

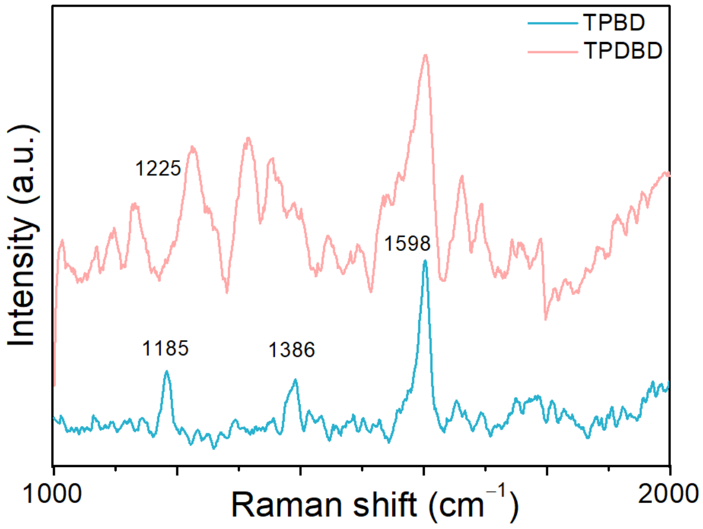

Supplementary Figure 7. Raman spectra of TPBD and TPDBD measured at 532 nm and 25 ± 1 °C.

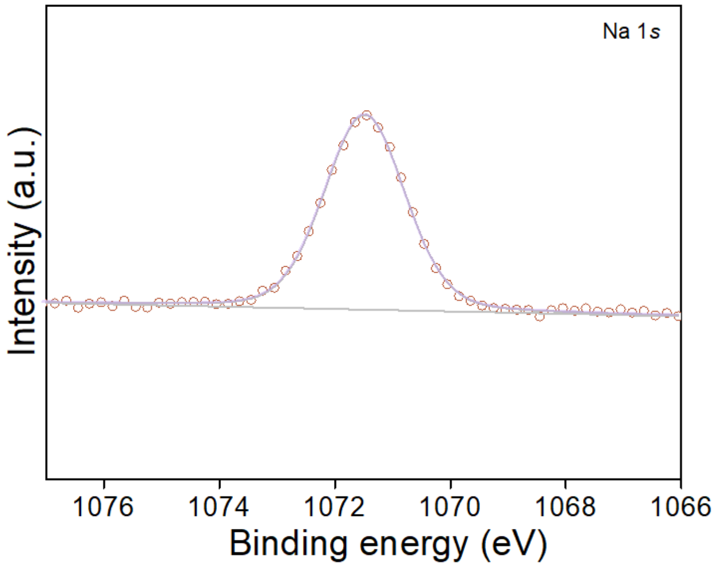

Supplementary Figure 8. XPS spectrum of Na 1*s* for TPDBD-CNa uncycled material tested at 25 ± 1 °C.

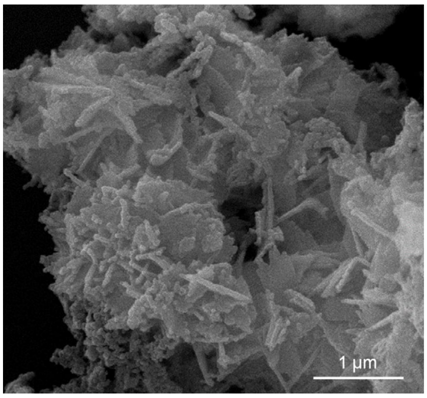

Supplementary Figure 9. SEM image of TPBD powder.

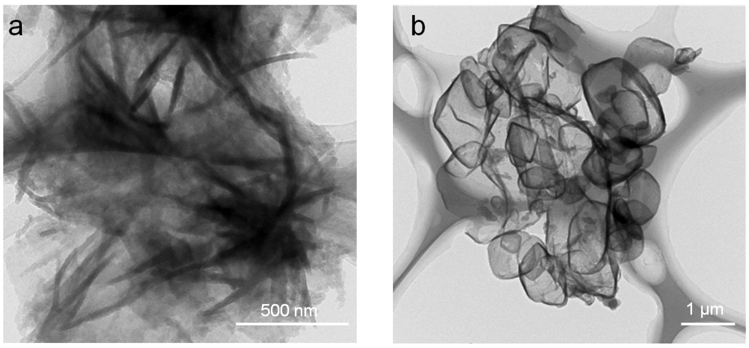

Supplementary Figure 10. TEM images of TPBD powder (a) and TPDBD powder (b).

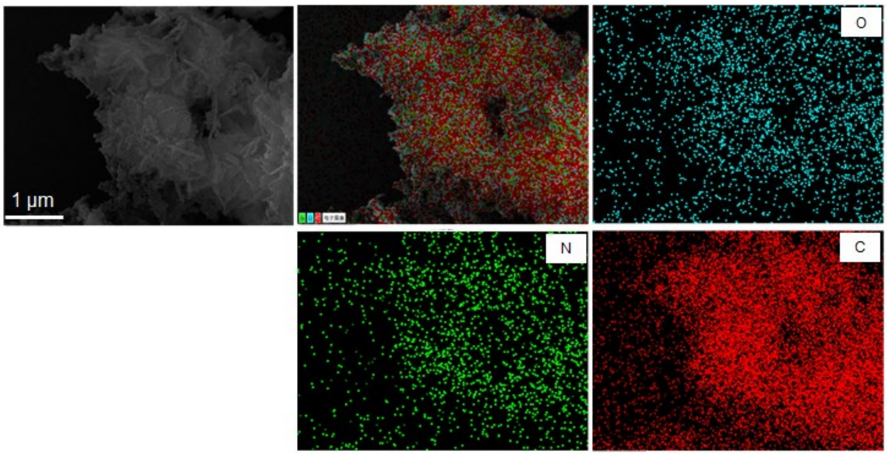

Supplementary Figure 11. SEM image and energy dispersive spectroscopy (EDS) images of TPBD powder.

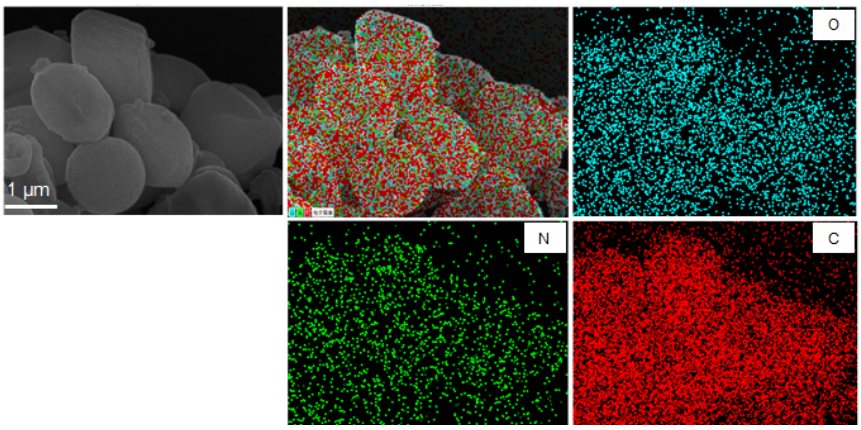

Supplementary Figure 12. SEM image and EDS images of TPDBD powder.

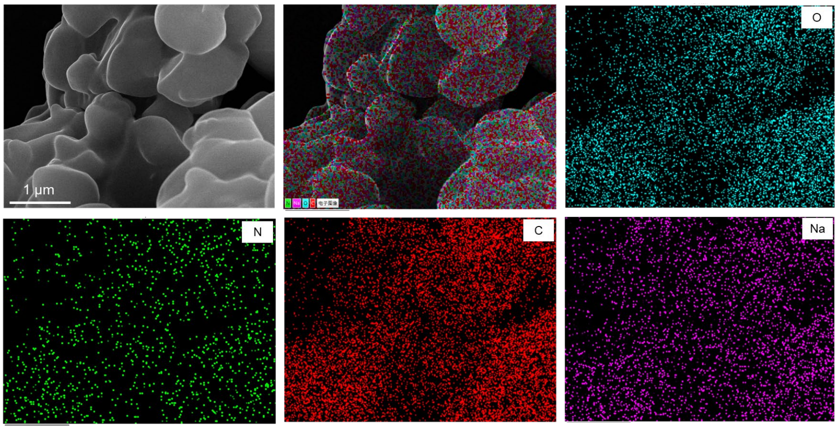

Supplementary Figure 13. SEM and EDS images of TPDBD-CNa powder.

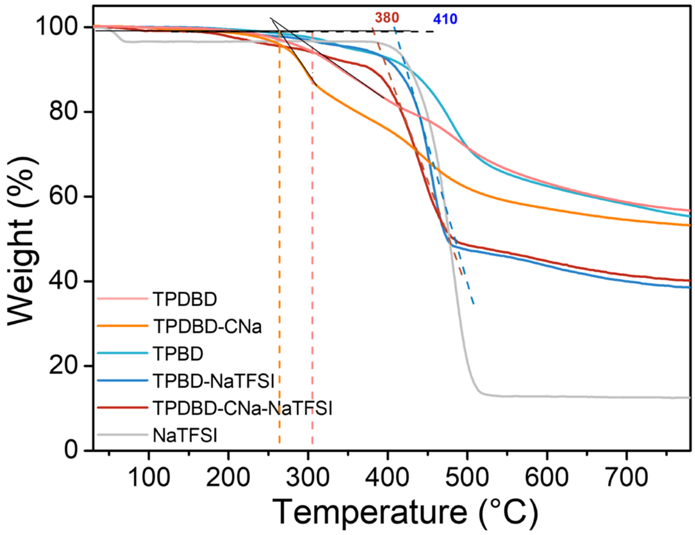

Supplementary Figure 14. TGA curves of TPDBD, TPDBD-CNa, TPBD, TPDBD-CNa-NaTFSI, TPBD-NaTFSI, and NaTFSI under N_2 atmosphere.

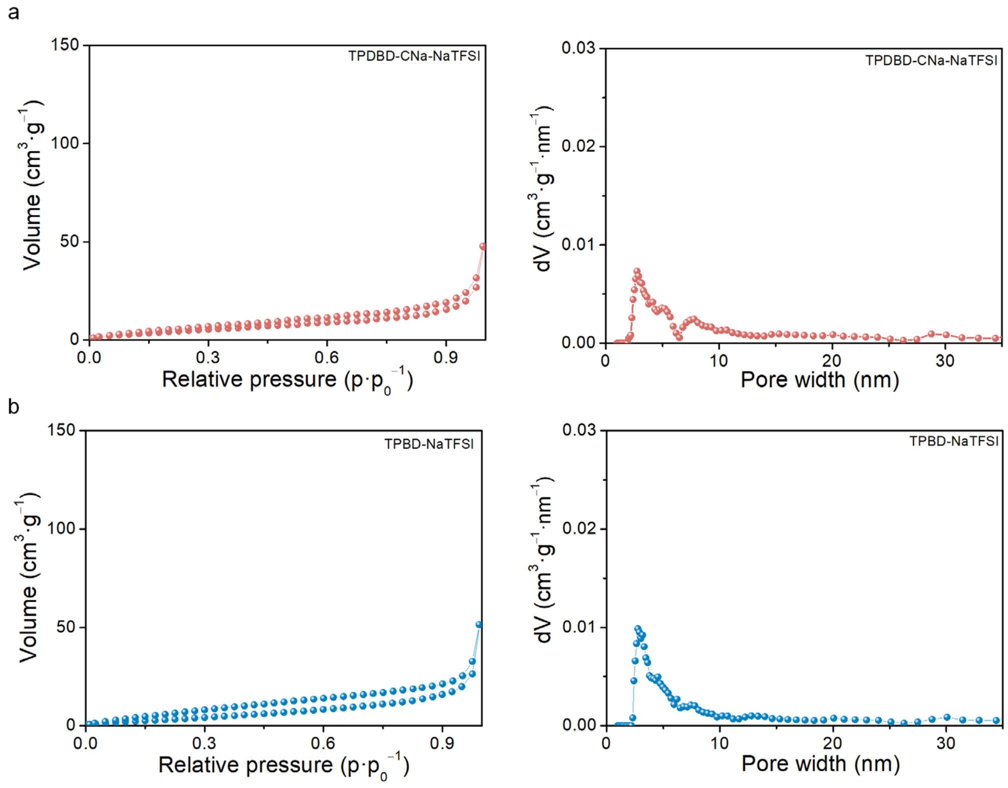

Supplementary Figure 15. N_2 adsorption and desorption curves and pore size distributions of TPDBD-CNa-NaTFSI (a) and TPBD-NaTFSI (b).

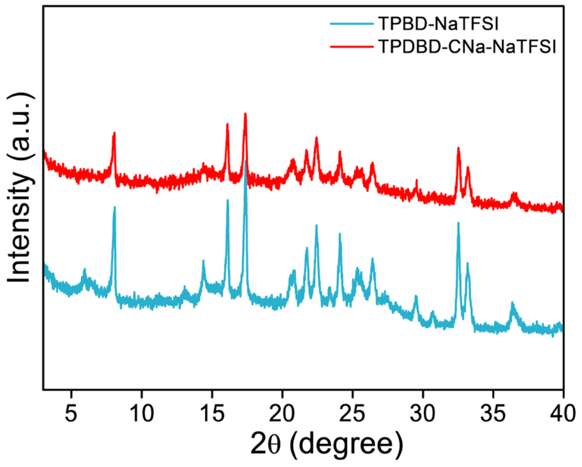

Supplementary Figure 16. PXRD patterns for TPBD-NaTFSI and TPDBD-CNa-NaTFSI.

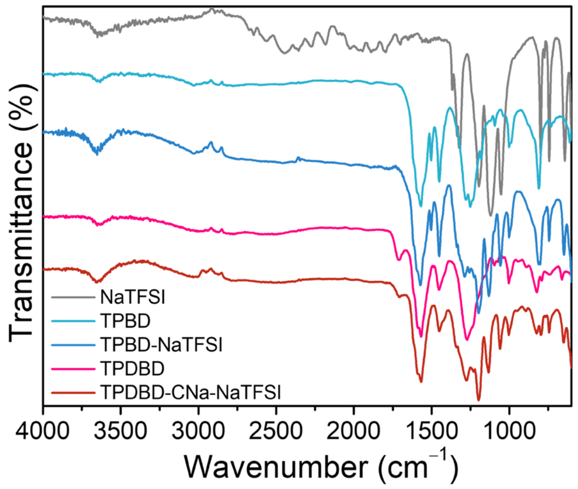

Supplementary Figure 17. FTIR spectra of TPDBD-CNa-NaTFSI, TPDBD, TPBD-NaTFSI, TPBD, and NaTFSI tested at 25 ± 1 °C.

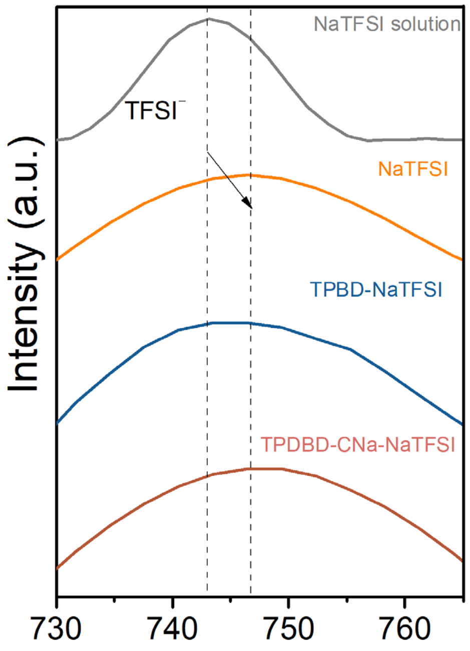

Supplementary Figure 18. Raman spectra of TPDBD-CNa-NaTFSI, TPBD-NaTFSI, NaTFSI, and NaTFSI solution tested at 25 ± 1 °C.

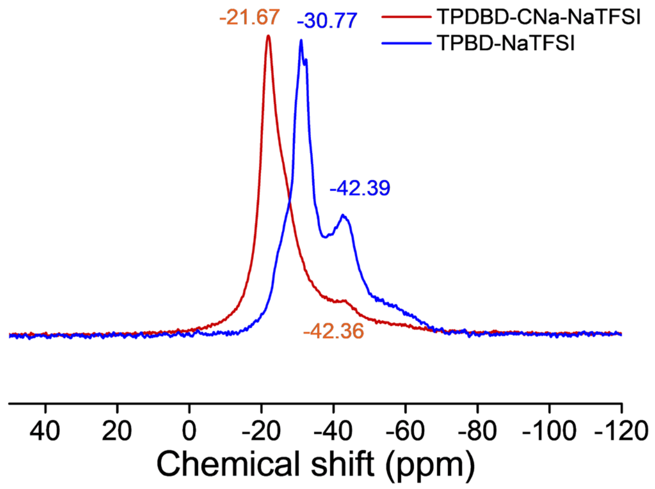

Supplementary Figure 19. ^{23}Na MAS NMR spectra of TPDBD-CNa-NaTFSI and TPBD-NaTFSI.

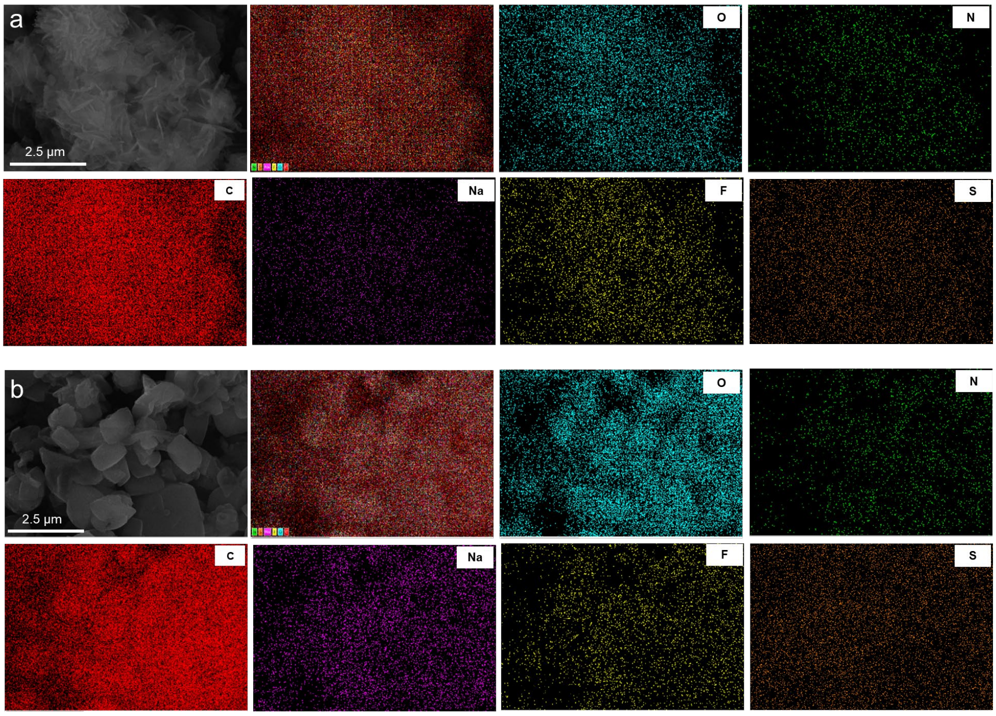

Supplementary Figure 20. (a) SEM image and EDS images of TPBD-NaTFSI powder. (b) SEM image and EDS images of TPDBD-CNa-NaTFSI powder.

tested at 25 ± 1 °C.

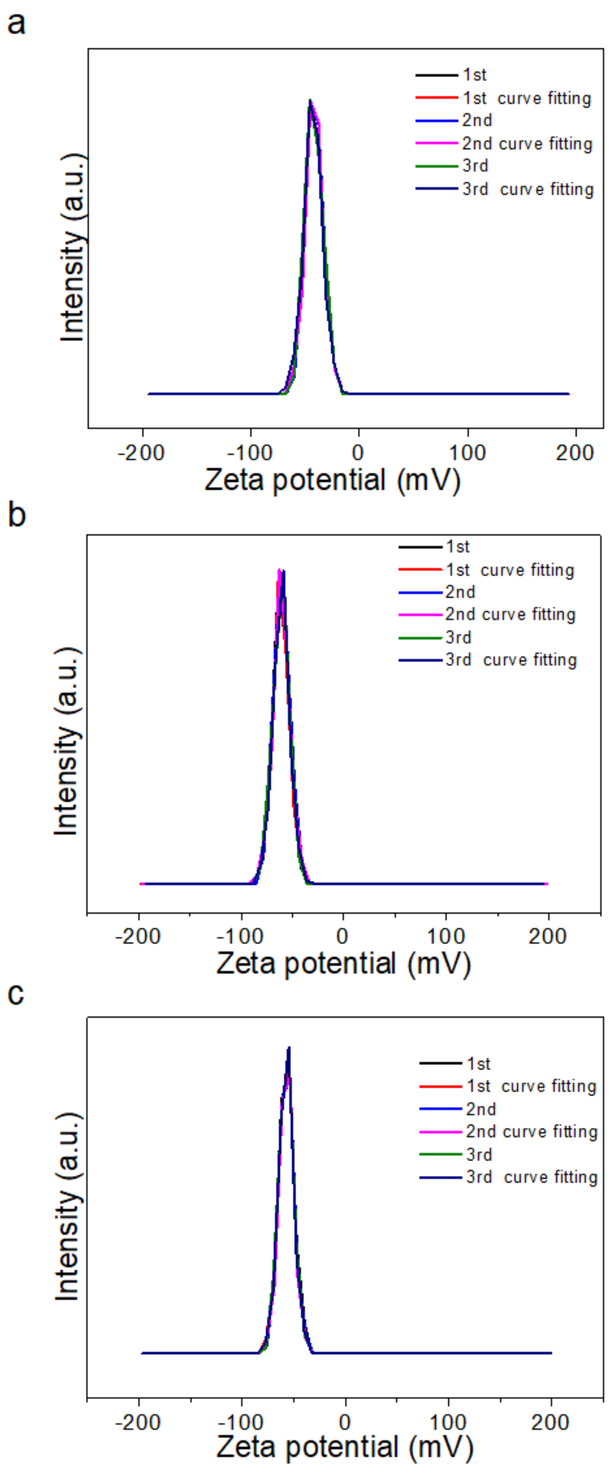

Supplementary Figure 21. Zeta potential results of TPBD (a), TPDBD (b), and TPDBD-CNa (c) tested at 25 ± 1 °C.

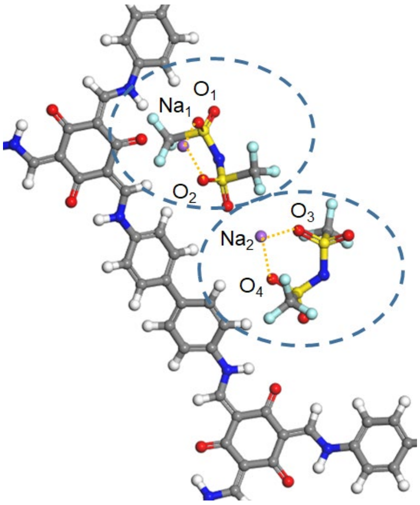

Supplementary Figure 22. Optimized coordination structure between NaTFSI and TPBD (the white, blue, gray, cyan, yellow, purple, and red spheres denote hydrogen, nitrogen, carbon, fluorine, sulfur, sodium, and oxygen, respectively).

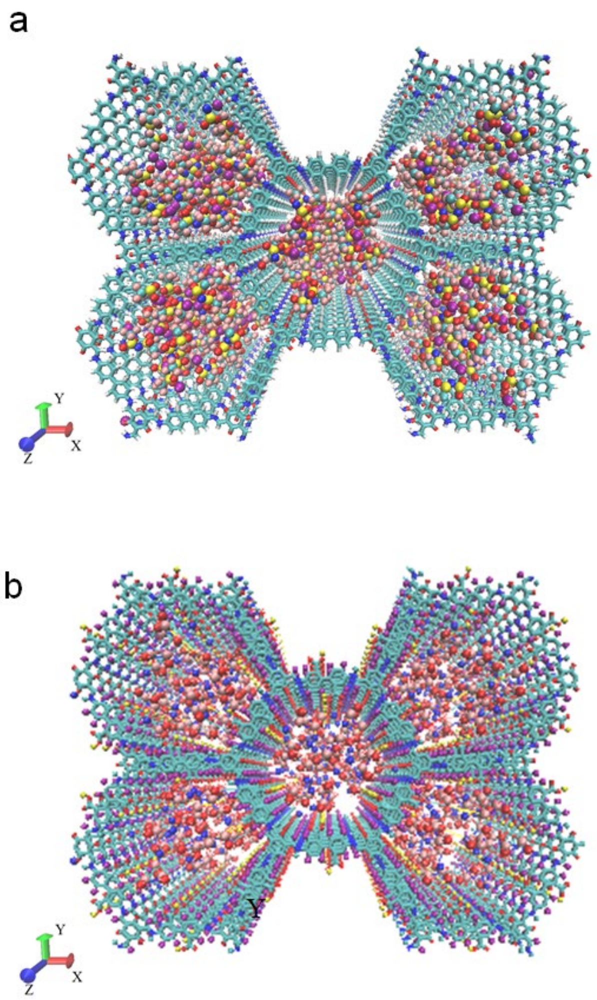

Supplementary Figure 23. MD simulations of TPBD-NaTFSI (a), and TPDBD-NaTFSI (b). The white, blue, cyan, pink, yellow, purple, and red spheres denote hydrogen, nitrogen, carbon, fluorine, sulfur, sodium, and oxygen atoms, respectively.

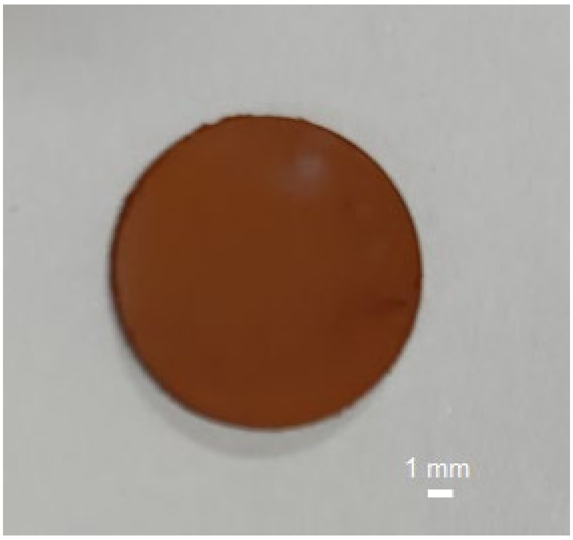

Supplementary Figure 24. Photo of SSE with ~9 wt.% solvents (propylene carbonate (PC) with 5% fluoroethylene carbonate (FEC)) in an argon-filled glove box (H_2O and O_2 <1 pmm) (No-flowing liquid on the surface).

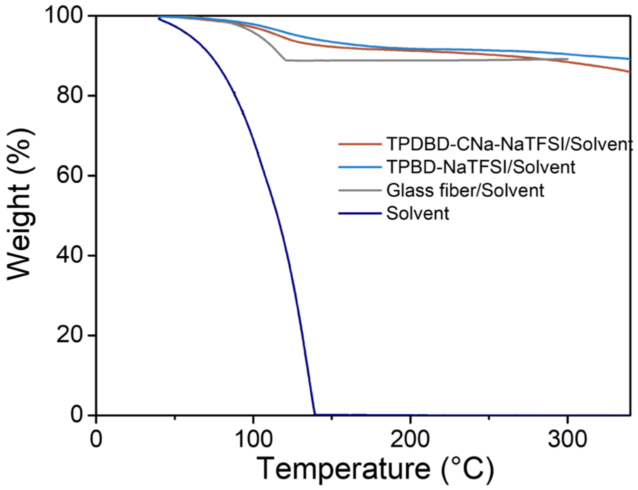

Supplementary Figure 25. TGA curves of the COF-based membranes and glass fiber with solvent (propylene carbonate (PC) with 5% fluoroethylene carbonate (FEC)) under N_2 atmosphere.

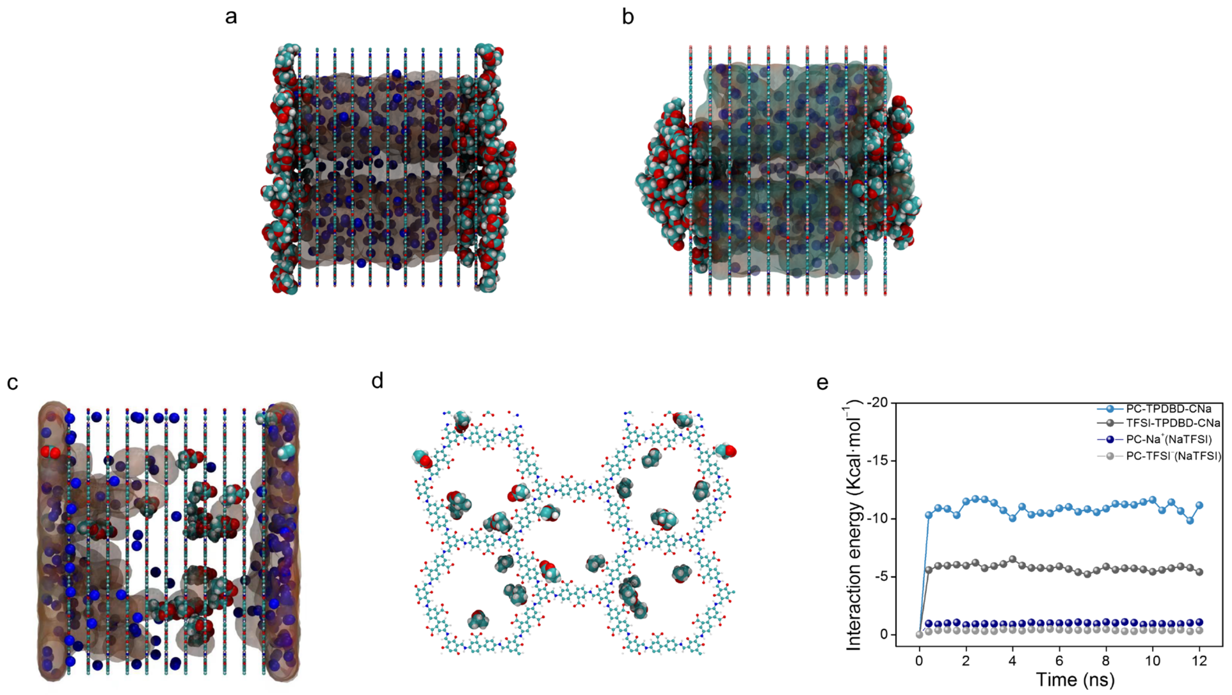

Supplementary Figure 26. (a) Equilibrium simulation of TPDBD-CNa-NaTFSI and PC. (b) Equilibrium simulation of TPBD-NaTFSI and PC. (c) Schematic of molecular simulations of TPDBD-CNa-NaTFSI/PC solvent (the white, blue, cyan, red, and purple spheres denote hydrogen, nitrogen, carbon, oxygen, and sodium, respectively. Brown area reprensts anion $TFSI^{-}$). (d) MD simulation of PC distribution. (e) Interaction energy between TPDBD-CNa and PC, and $TFSI^{-}$ in TPDBD-CNa-NaTFSI/PC solvent, and interaction energy between PC and Na^{+}, and $TFSI^{-}$ of (NaTFSI).

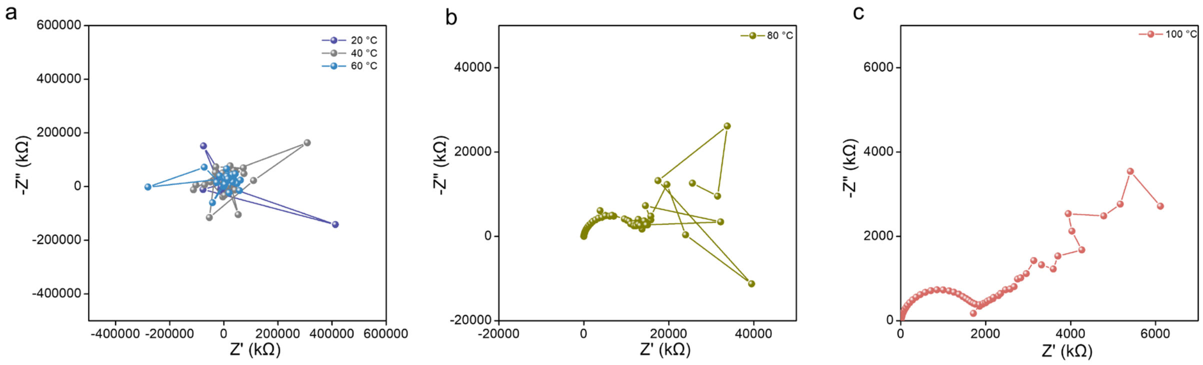

Supplementary Figure 27. (a) EIS of blocking titanium Ti|TPDBD-CNa-NaTFSI|Ti symmetric cells from 25 to 100 ºC without solvent. (b) EIS of Ti|TPDBD-CNa-NaTFSI|Ti symmetric cells at 80 ºC without solvent. (c) EIS of Ti|TPDBD-CNa-NaTFSI|Ti symmetric cells at 100 ºC without solvent.

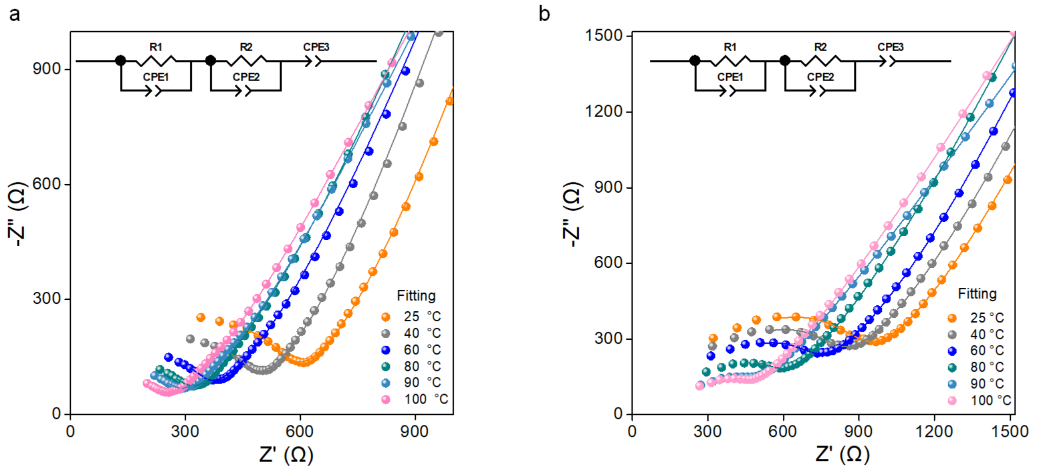

Supplementary Figure 28. EIS profiles measured at various temperatures from 25 to 100 ºC of Ti|TPDBD-CNa-QSSE|Ti symmetric cell (a) and Ti|TPBD-QSSE|Ti symmetric cell (b), and fitted by equivalent circuit.

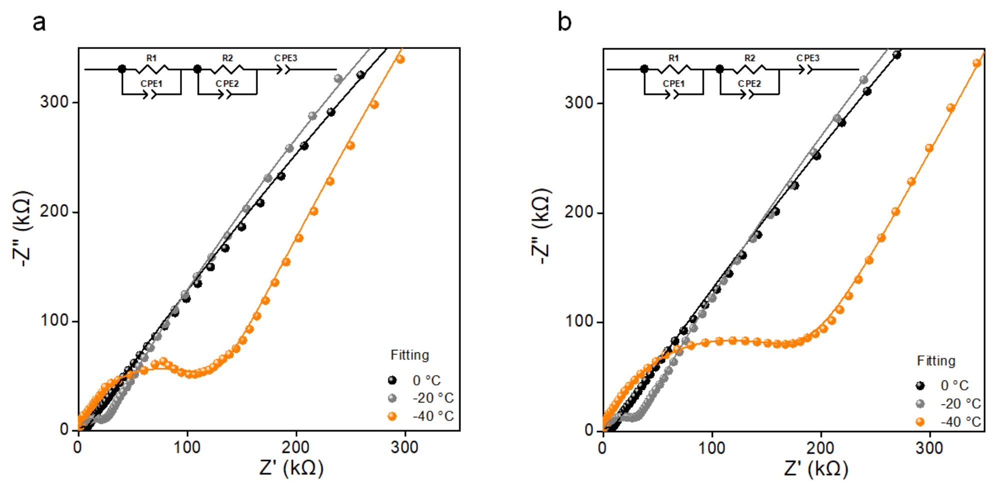

Supplementary Figure 29. EIS profiles measured at various temperatures from 0 to –40 ºC of Ti|TPDBD-CNa-QSSE|Ti symmetric cell (a) and Ti|TPBD-QSSE|Ti symmetric cell (b), and fitted by equivalent circuit.

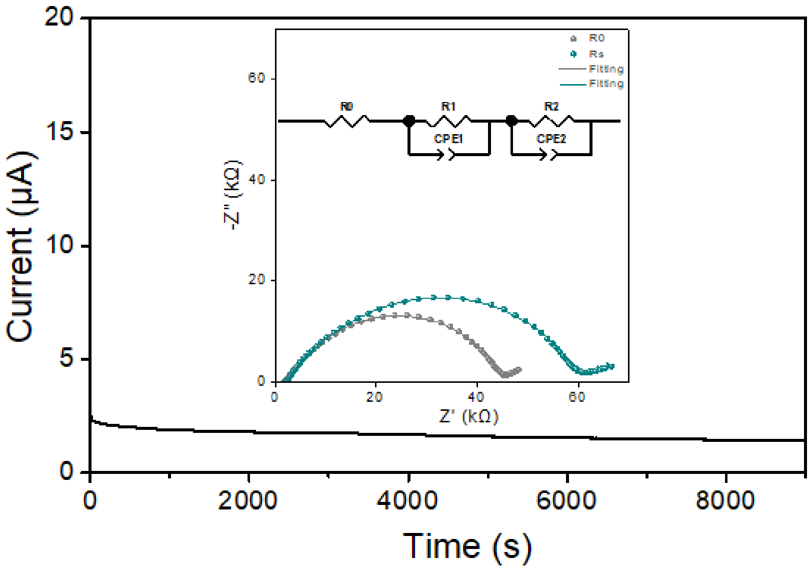

Supplementary Figure 30. Current-time curve of Na|TPBD-QSSE|Na symmetric cell (the inset of EIS at initial and steady states).

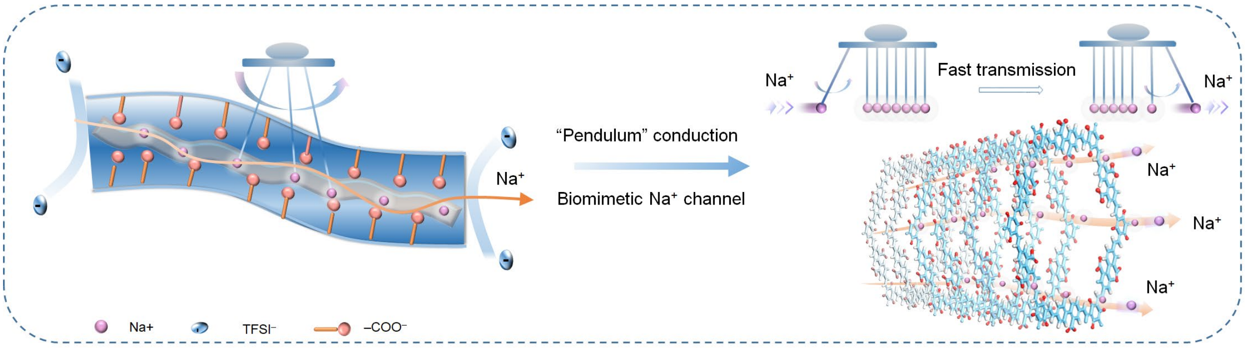

Supplementary Figure 31. Pendular Na^+ transport mechanism in the biomimetic Na^+ channel (red spheres denote oxygen, and cyan sticks denote covalent organic framework).

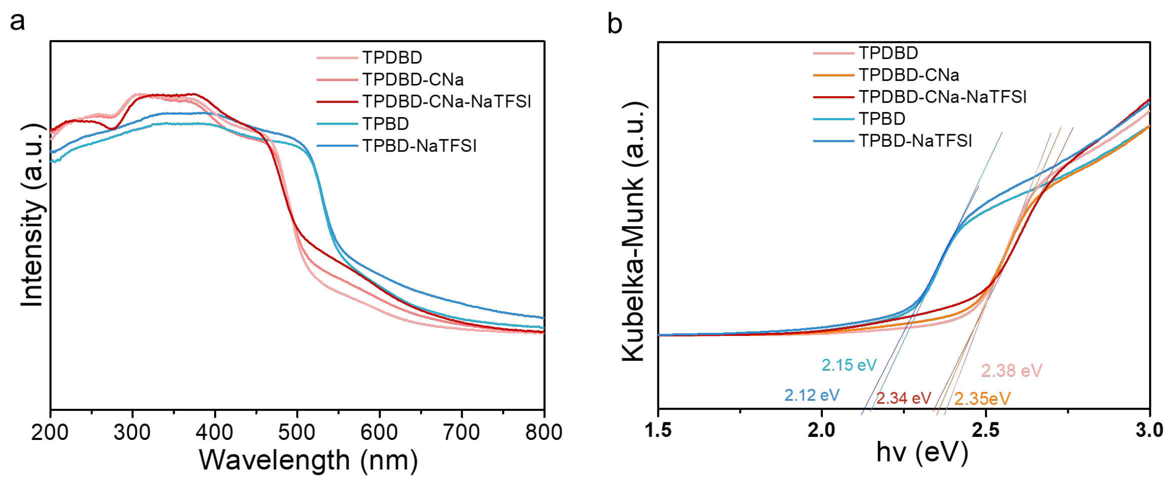

Supplementary Figure 32. (a) UV-vis absorption spectra of TPDBD, TPDBD-CNa, TPDBD-CNa-NaTFSI, TPBD, and TPBD-NaTFSI tested at 25 ± 1 °C. (b) Tauc plots of prepared structures.

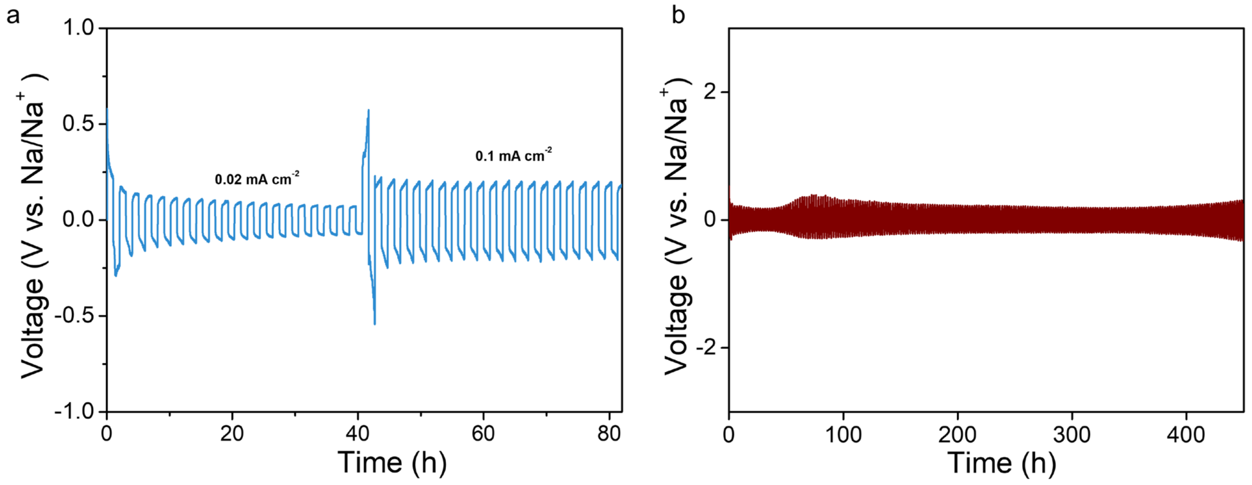

Supplementary Figure 33. (a) Na stripping-plating test of Na|TPDBD-CNa-QSSE|Na at 25 ± 1 °C for 2 h per cycle. (b) Na stripping-plating test of Na|TPDBD-CNa-QSSE|Na at current density of 0.05 mA cm^{-2} and 25 ± 1 °C for 450 h and 2 h per cycle.

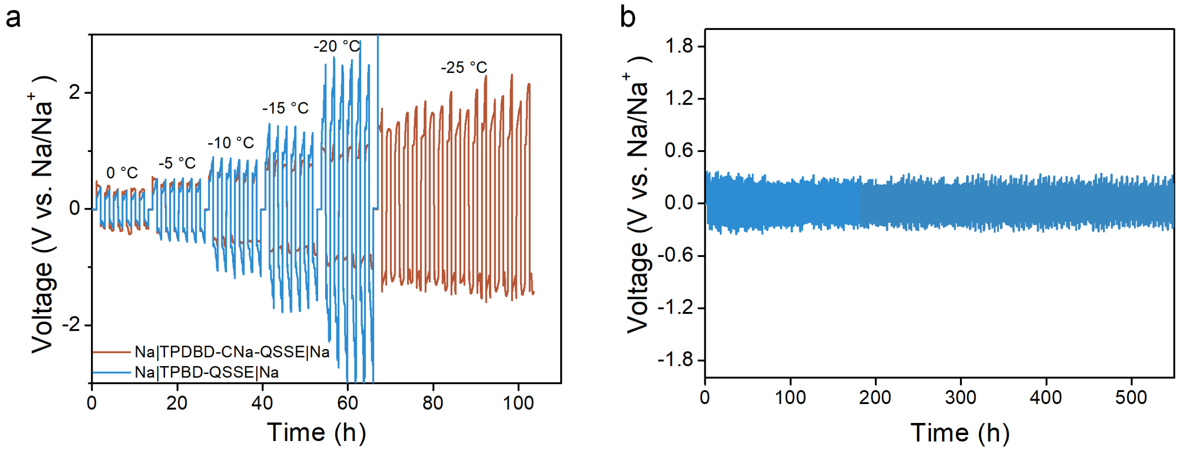

Supplementary Figure 34. (a) Temperature-dependent galvanostatic cycling of Na stripping-plating test of Na|QSSEs|Na symmetric cell at 0, –5, –10, –15, –20, and –25 °C with a current density of 0.01 mA cm^{-2}. (b) Long cycle performance of Na|TPDBD-CNa-QSSE|Na symmetric cell at 0 °C with a current density of 0.01 mA cm^{-2}.

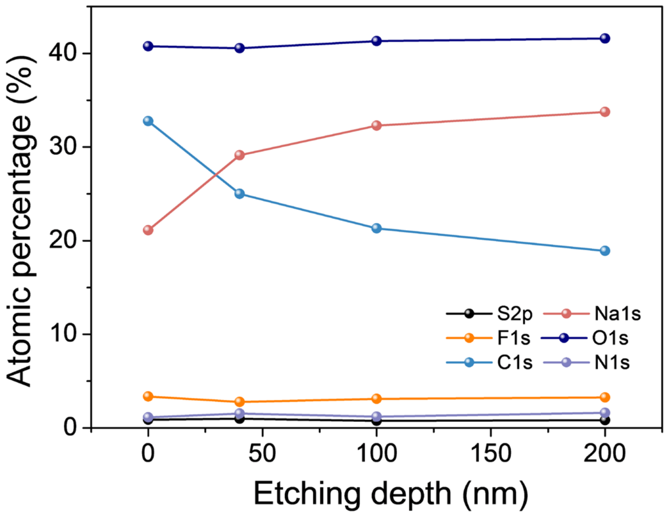

Supplementary Figure 35. Atomic ratio of the S, Na, F, O, C, and N elements in the SEI at various sputtering depths of the cycled Na anode surface composition in Na|TPDBD-CNa-QSSE|Na symmetric cell over 20 cycles at 0.02 mA cm^{-2} and 25 ± 1 °C.

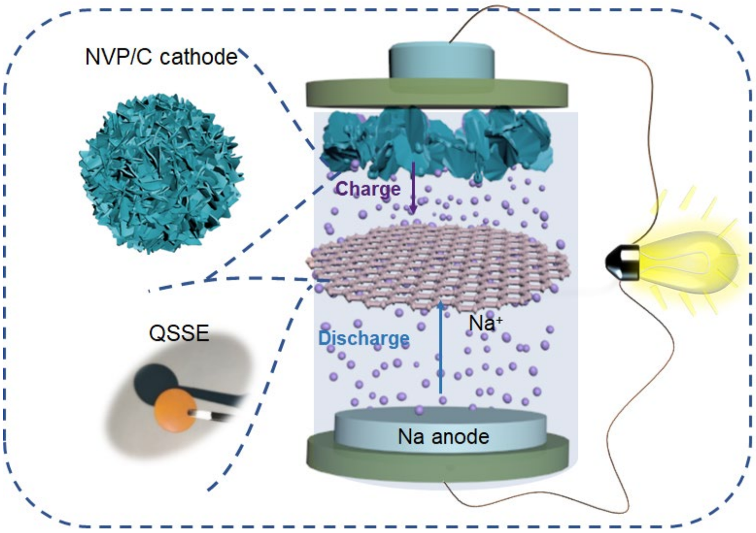

Supplementary Figure 36. Schematic diagram of SSBs assembled with COF electrolyte and NVP/C cathode material (the purple spheres denote sodium).

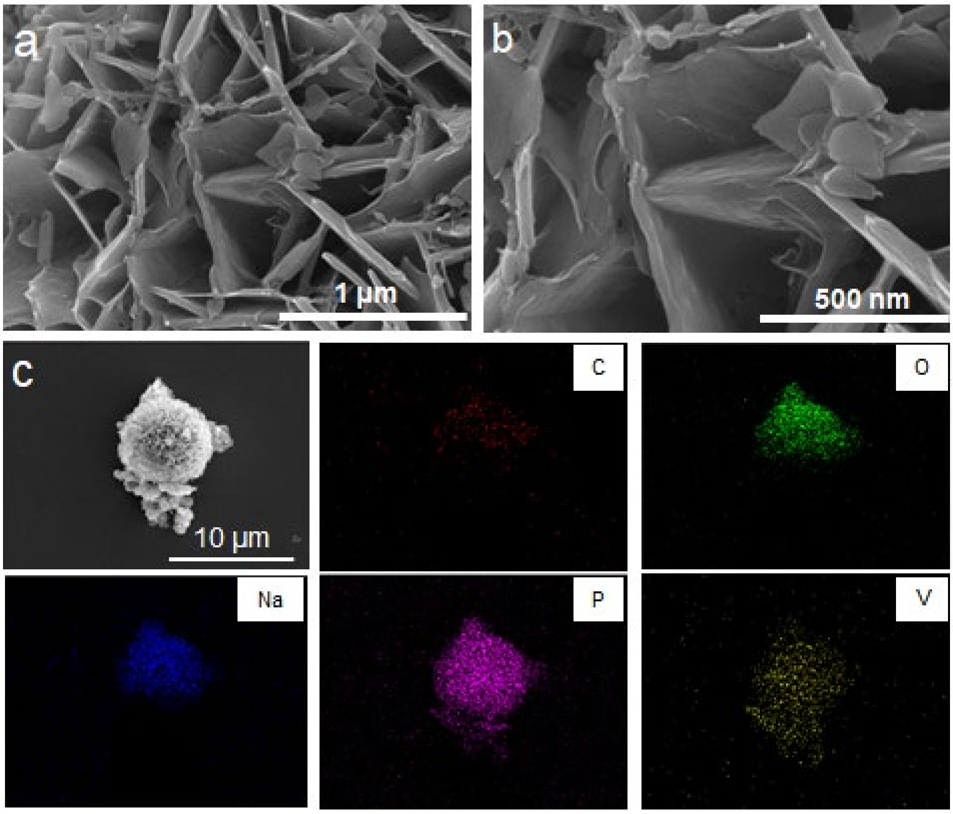

Supplementary Figure 37. (a-b) SEM images of NVP/C powder under different magnifications. (c) SEM image and EDS images of NVP/C.

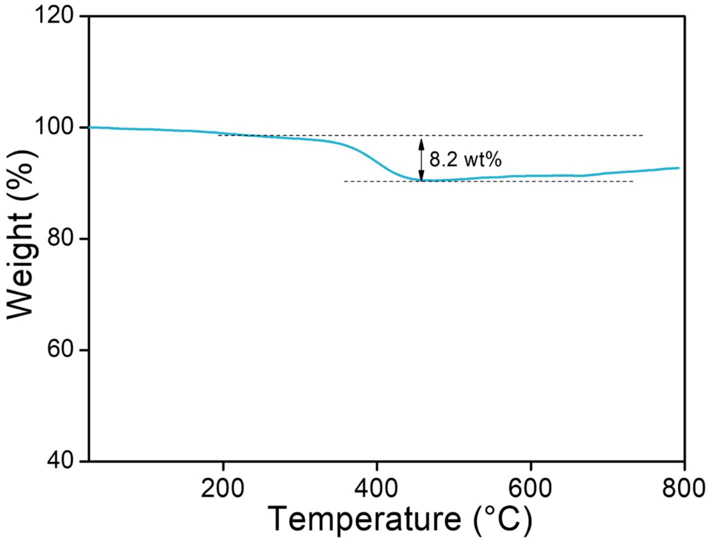

Supplementary Figure 38. TGA curve of the NVP/C powder measured under air flow. The TGA profile of NVP/C indicates 8.2 wt.% carbon in the NVP/C.

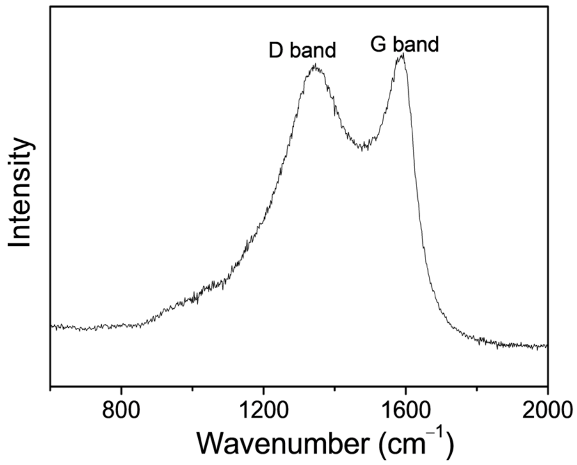

Supplementary Figure 39. Raman spectrum of NVP/C powder. Raman spectrum of NVP/C displays two characteristic bands located at 1340 cm^{-1} (D-band, disorder carbon) and 1588 cm^{-1} (G-band, crystalline graphitized carbon), and the peak intensity ratio of the D to G band (I_D/I_G) is 0.97, demonstrating a relatively high degree of graphitization of the carbon layer.

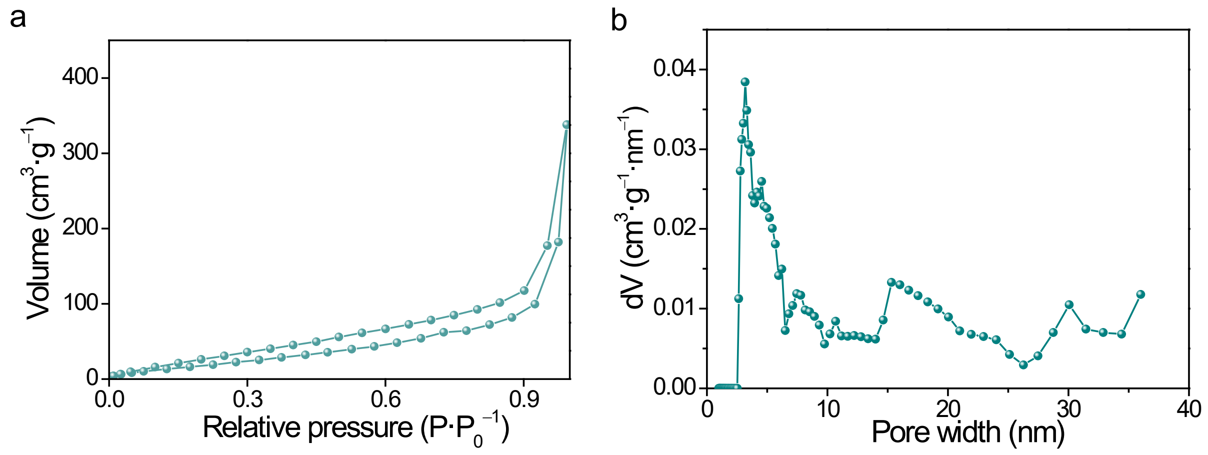

Supplementary Figure 40. N_2 adsorption and desorption curve (a) and pore size distribution of NVP/C (b). The NVP/C exhibits BET surface area of 76 m^2 g^{-1} and total pore volume of 0.52 cm^3 g^{-1}, and its hierarchical porous structure could facilitate the insertion and extraction of Na^+.

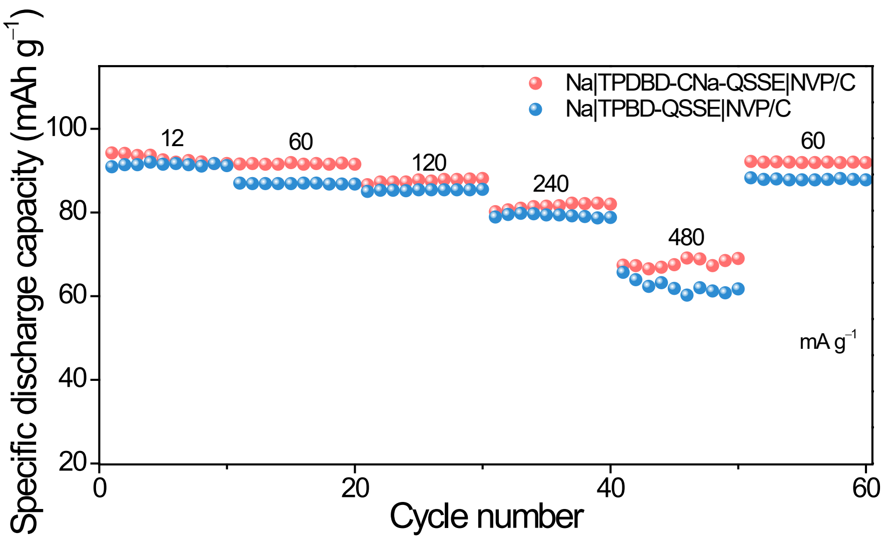

Supplementary Figure 41. Rate performances of Na|TPDBD-CNa-QSSE|NVP/C and Na|TPBD-QSSE|NVP/C cell tested at 25 ± 1 °C.

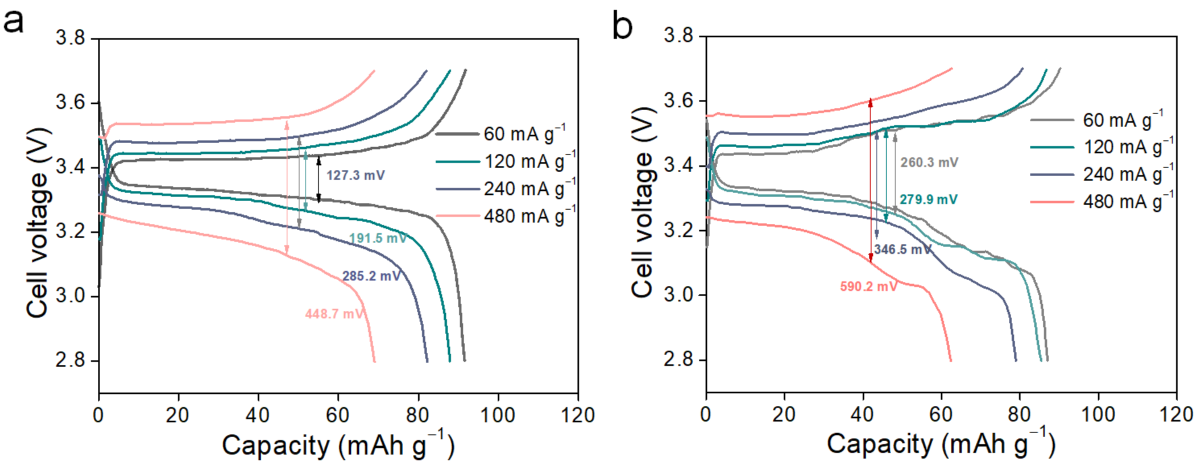

Supplementary Figure 42. (a) Voltage profiles of Na|TPDBD-CNa-QSSE|NVP/C at different specific currents and 25 ± 1 °C. (b) Voltage profiles of Na|TPBD-QSSE|NVP/C at different specific currents and 25 ± 1 °C.

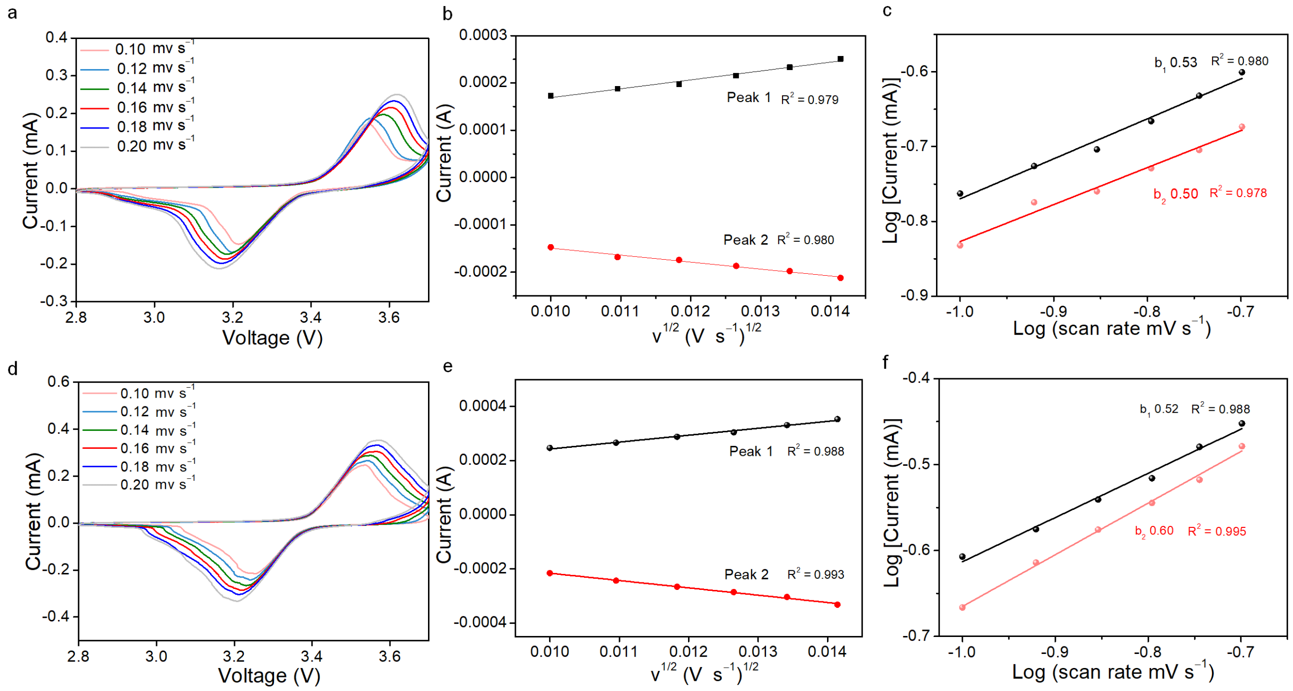

Supplementary Figure 43. (a) CV curves of Na|TPBD-QSSE|NVP/C cell, (b) corresponding relationship between peak current and square root of sweep rate ($v^{1/2}$), and (c) linear fitting plots of the transformed peak current versus scan rate. (d) CV curves of Na|TPDBD-CNa-QSSE|NVP/C cell, (e) corresponding relationship between peak current and square root of sweep rate ($v^{1/2}$), and (f) linear fitting plots of the transformed peak current versus scan rate. All the electrochemical tests were carried out at 25 ± 1 °C.

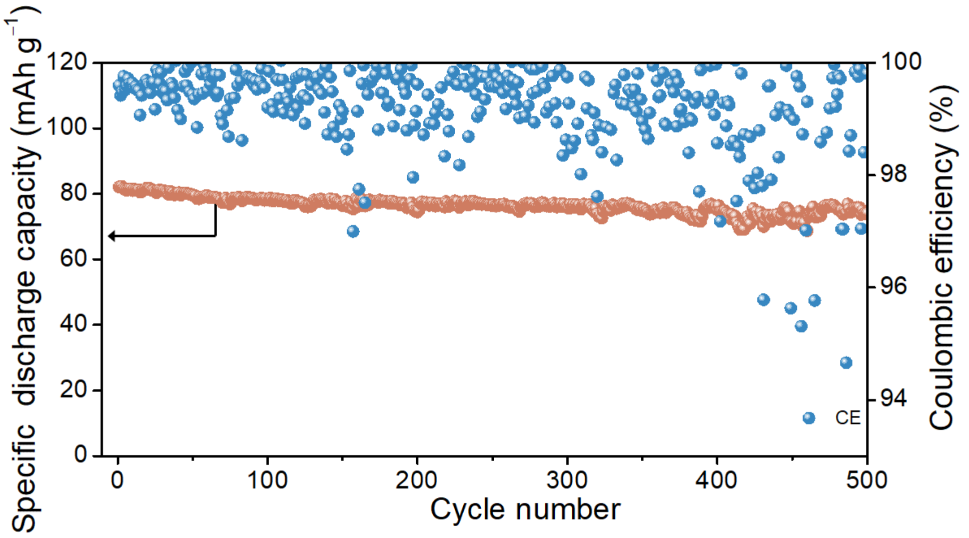

Supplementary Figure 44. Cycling stability of Na|TPDBD-CNa-QSSE|NVP/C cell at 120 mA g^{-1} and 25 ± 1 °C.

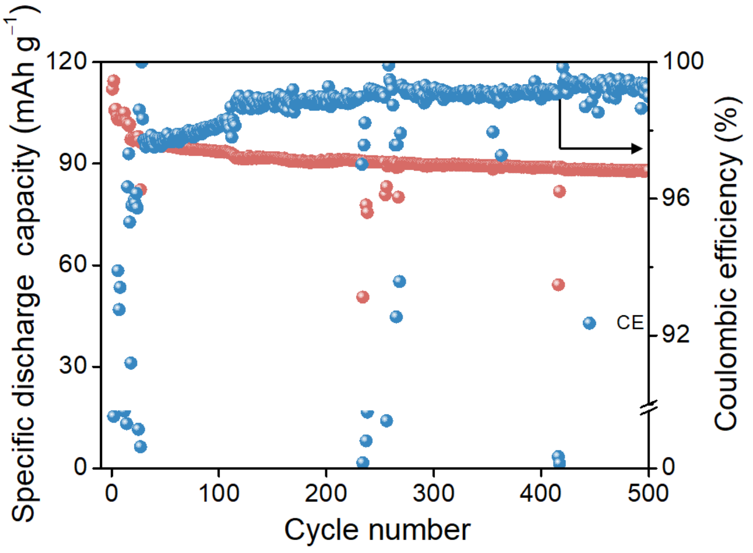

Supplementary Figure 45. Cycling stability of Na|TPBD-QSSE|NVP/C cell at 60 mA g^{-1}.

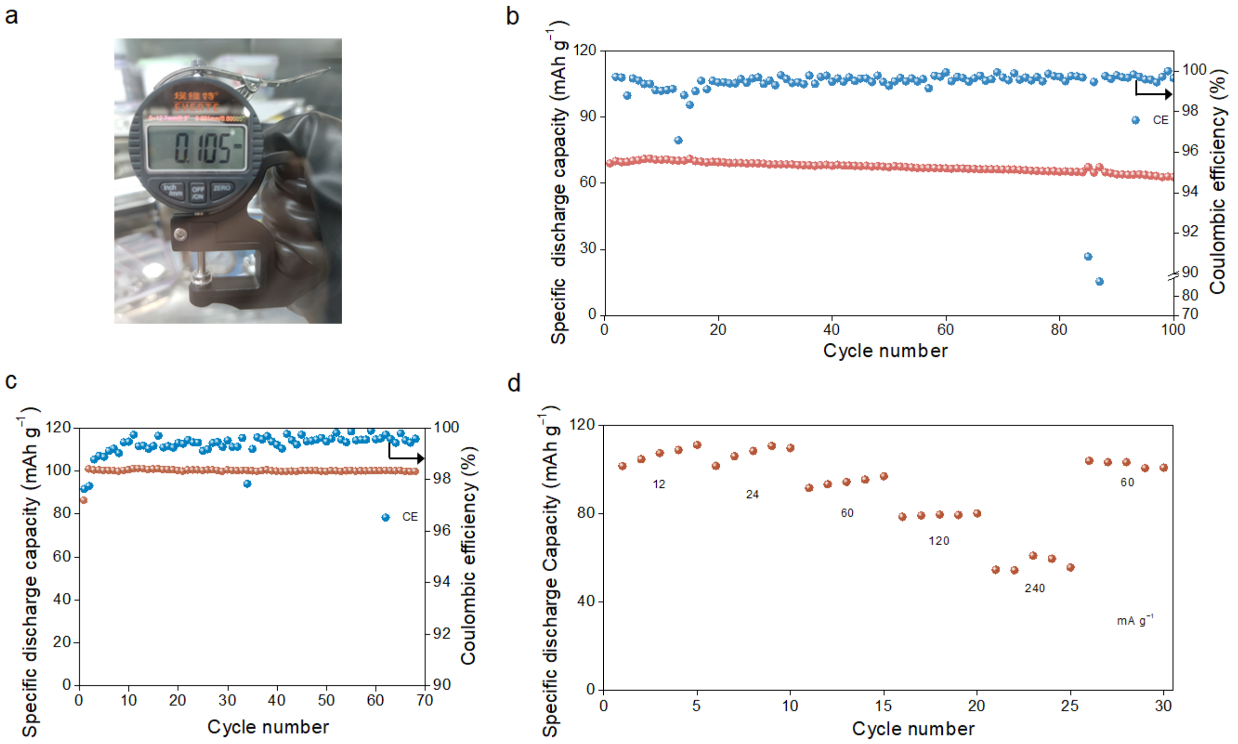

Supplementary Figure 46. (a) Photo of Na anode thickness. (b) Cycle stability at 60 mA g^{-1}, (c) cycle stability at 12 mA g^{-1}, and (d) rate performance of the Na|TPDBD-CNa-QSSE|NVP/C with high mass loading of active materials (2.6 mg cm^{-2}) in the cathode. All the electrochemical tests were carried out at 25 ± 1 °C.

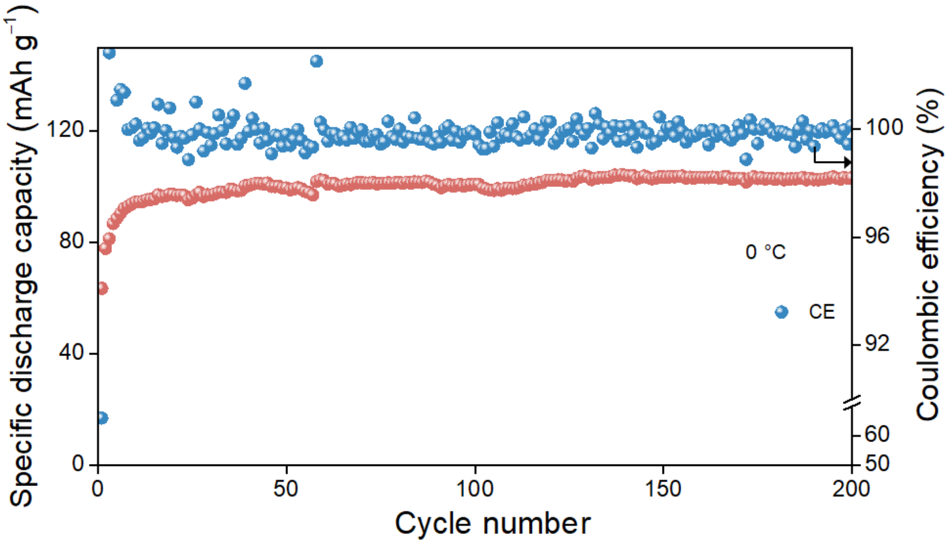

Supplementary Figure 47. Cycle performance of Na|TPDBD-CNa-QSSE|NVP/C cell at 12 mA g^{-1} and 0 °C.

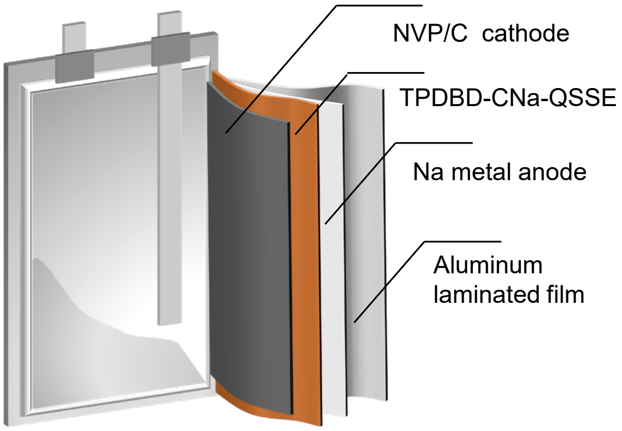

Supplementary Figure 48. Schematic of the bendable Na|TPDBD-CNa-QSSE|NVP/C pouch cell.

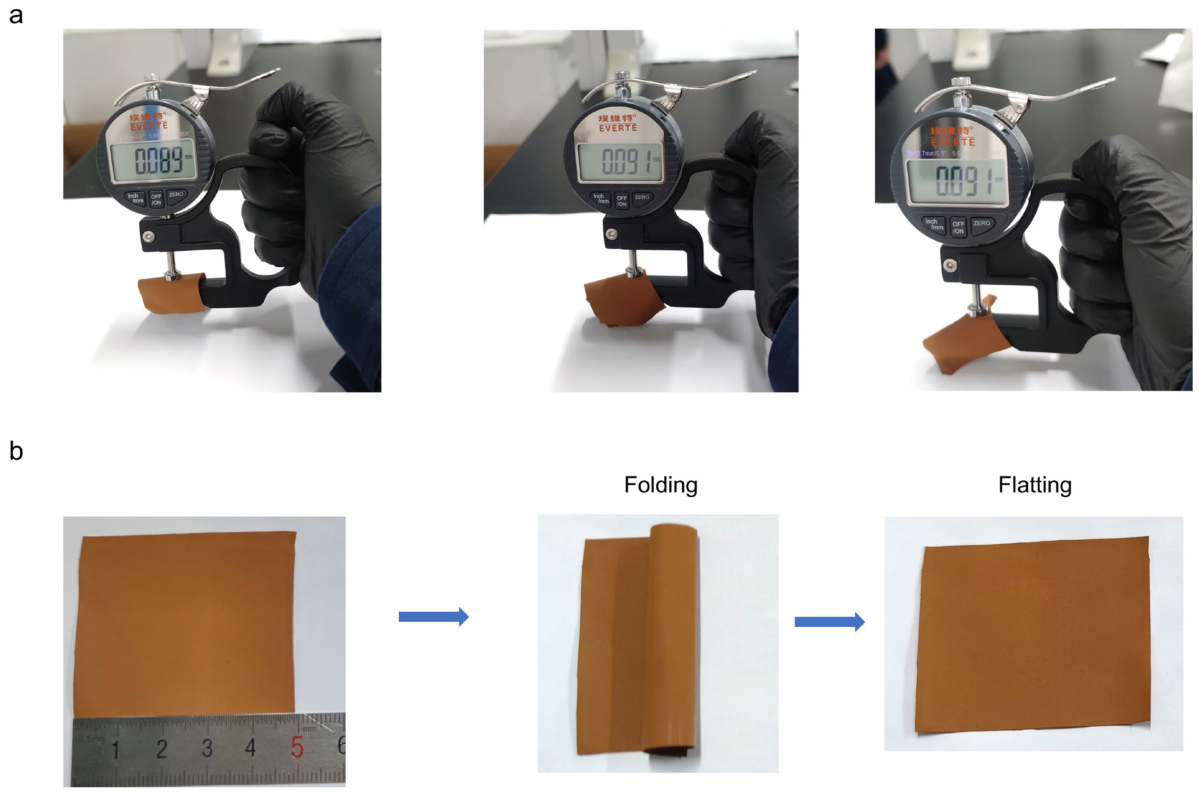

Supplementary Figure 49. (a) Photos of the TPDBD-CNa membrane thickness at different positions carried out in air. (b) Photos of flexible freestanding solid TPDBD-CNa membrane.

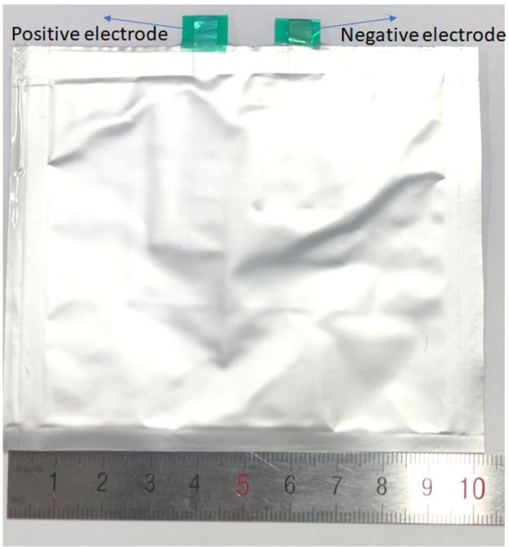

Supplementary Figure 50. Photo of a quasi-solid-state pouch cell with single electrode layer.

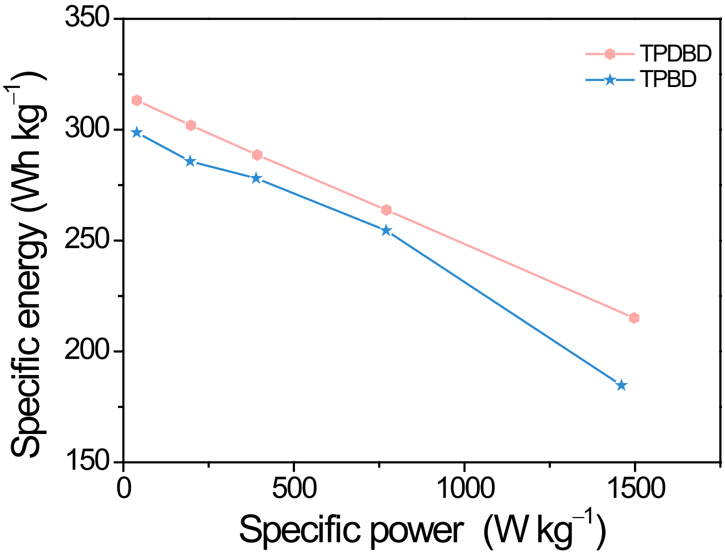

Supplementary Figure 51. Ragone plots of Na|TPDBD-CNa-QSSE|NVP/C cell and Na|TPBD-QSSE|NVP/C cell based on the mass of the positive electrode active material.

Supplementary Table 1. Elemental analysis (EA) and inductive coupled plasma emission spectrometer (ICP) of TPBD, TPDBD, TPDBD-CNa, TPDBD-NaTFSI, and TPBD-NaTFSI.

Samples	Mass percentage (wt.%)					
	N	C	H	S	O	Na
TPBD	9.15	70.66	5.08	-	15.12	-
TPDBD	8.02	56.89	5.21	-	29.86	-
TPDBD-CNa	7.05	51.42	4.87	-	32.76	3.80
TPBD-NaTFSI	7.36	46.32	3.10	8.31	25.35	3.07
TPDBD-NaTFSI	6.30	35.13	2.82	7.15	31.09	5.46

Note: The measurement error of EA ≤0.1%, and the measurement error of ICP ≤0.5%.

Supplementary Table 2. Summary of bulk resistance, fitting error, and Chi-Squared of Ti|QSSEs|Ti symmetric cells (Supplementary Figures 28-29) at different temperatures.

	Resistance (Ω)		Error (%)		Chi-Squared	
Temperature (°C)	TPDBD-CNa-QSSE	TPBD-QSSE	TPDBD-CNa-QSSE	TPBD -QSSE	TPDBD-CNa-QSSE	TPBD-QSSE
25	271.7	390.8	6.47	2.38	$3.06{\times}10^{-5}$	$2.42{\times}10^{-5}$
40	157.6	303.3	9.83	2.33	$3.82{\times}10^{-5}$	$1.92{\times}10^{-5}$
60	125.3	219.2	6.22	2.40	$1.49{\times}10^{-4}$	$1.55{\times}10^{-5}$
80	91.58	121.7	5.36	4.45	$8.33{\times}10^{-5}$	$1.57{\times}10^{-5}$
90	66.73	89.94	8.59	9.62	$7.76{\times}10^{-5}$	$8.64{\times}10^{-5}$
100	45.14	62.77	9.10	6.62	$7.77{\times}10^{-5}$	$1.88{\times}10^{-5}$
0	457	811.6	9.45	6.54	$7.79{\times}10^{-5}$	$8.48{\times}10^{-5}$
-20	1994	5110	9.65	7.59	$2.22{\times}10^{-3}$	$3.56{\times}10^{-4}$
-40	3943	8703	9.86	9.38	$1.37{\times}10^{-3}$	$4.33{\times}10^{-4}$

Supplementary Table 3. Summary of resistance, fitting error, and Chi-Squared of Na|QSSEs|Na symmetric cells (Figure 5e and Supplementary Figure 30).

		Resistance (Ω)	Error (%)	Chi-Squared
TPDBD-CNa-QSSE	R_{10}	217.4	8.32	3.18×10^{-4}
	R_{20}	17701	0.49	
	R_{1s}	358.5	5.88	3.00×10^{-3}
	R_{2s}	19481	1.17	
TPBD-QSSE	R_{10}	1848	4.17	8.20×10^{-4}
	R_{20}	41467	1.78	
	R_{1s}	4490	7.03	2.20×10^{-4}
	R_{2s}	53932	2.42	

Supplementary Table 4. Comparison of physicochemical parameters for various Li-ion and Na-ion solid-state electrolytes based on blocking symmetric cell.

Materials	Li^+/ Na^+	Non-aqueous liquid additive	Ion transference number	Electrochemical window (V)	Thickness (μm)	Ea (V)	σ (S cm^{-1})/ (°C)	Ref.
ICOF-2/PC	Li^+	55 wt.% PC	0.8	4	NA	0.24	3.05×10^{-5}/(25)	1
MIT-20-LiCl	Li^+	~70 wt.% PC	0.75	NA	NA	0.32	1.3×10^{-5}/(25)	2
$LiPF_6$@ ZIF-8	Li^+	45 wt.% EC/DMC/EMC	0.52	4.7	NA	0.458	1.05×10^{-5}/(25)	3
$LiPF_6$@PAF-1	Li^+	50 wt.% EC/DMC	0.859	4.9	NA	0.15	4×10^{-4}/(25)	4
3D-UIO-66/PAN/PEO/LiTFSI	Li^+	NA	0.52	NA	100	0.2	2.89×10^{-4}/(60)	5
NaOOC-COF	Na^+	10.0 μL (1.0 M $NaPF_6$ /PC)	0.9	4.2	200	0.24	2.68×10^{-4}/(25)	6
MIL-121/Na^+SE	Na^+	50 wt.% (1 M NaClO4 /PC)	NA	NA	1000	0.36	1.2×10^{-4}/(30)	7
PLM@LE	Na^+	90 μL ($NaClO_4$-PC with 5% FEC)	0.33	4.8	120	0.112	6.6×10^{-5}/(25)	8
TPDBD-CNa-QSSE	Na^+	~9 wt.% solvent	0.90	5.32	400	0.204	1.30×10^{-4}/(25)	This work

*Unless otherwise noted, tests were conducted at 25 °C.

Supplementary Table 5. Comparison of physicochemical parameters for various Li-ion and Na-ion solid-state electrolytes based on blocking symmetric cell measurements.

Materials	Li^+/ Na^+	Non-aqueous liquid additive	Ion transference number	Electrochemical window (V)	Thickness (μm)	Ea (V)	σ (S cm^{-1})/ (°C)	Ref.
Li^+/Al-Td-MOF-1	Li^+	~50 wt.% PC	NA	NA	NA	0.1	5.7×10^{-5}/(25)	9
UN-SLi	Li^+	33 wt.% EC/DEC	0.74	NA	NA	NA	1.38×10^{-5}/(25)	10
L@K/C	Li^+	NA	NA	4.2	7.1	NA	1.62×10^{-4}/(30)	11
CuBTC-PSS	Li^+	0.3 mg cm^{-2} (1M LiTFSI-PC)	NA	5.4	38	0.096	5.75×10^{-4}/(25)	12
COF-NUST 7-8	Li^+	NA	0.11	4.2	NA	0.273	1.09×10^{-5}/(40)	13
LGZ	Li^+	19.4 wt.% PC	0.885	4.0	100	NA	1.61×10^{-4}/(30)	14
LiOOC-COF3	Li^+	NA	0.91	4.2	200	0.17	1.36×10^{-5}/(25)	15
DLC	Li^+	NA	0.85	4.5	32	NA	1.65×10^{-4}/(23)	16
MIT-20-Na	Na^+	Infiltrating with PC	NA	NA	NA	0.39	1.8×10^{-5}/(25)	2
NaFNFSI/PEO	Na^+	NA	0.24	4.87	150	NA	3.36×10^{-4}/(80)	17
NaPTAB-SGPE	Na^+	66 wt.% PC	0.91(60)	5.2	50	0.127 (60)	9.4×10^{-5}/(25)	18
Ge-COF-Na	Na^+	PC	NA	NA	NA	0.25	3.4×10^{-5}/(100)	19
TPDBD-CNa-QSSE	Na^+	~9 wt.% solvent	0.90	5.32	400	0.204	1.30×10^{-4}/(25)	This work

*Unless otherwise noted, tests were conducted at 25 °C.

Supplementary Table 6. Comparison of symmetrical cells and full cells performance (based on Li/Na metal negative electrode) for the reported Li^+/Na^+ SSEs.

Materials	Li^+/ Na^+	Non-aqueous liquid additive	Symmetrical cells: Current densities (mA cm^{-2})	Symmetrical cells: Time (h)	Full cells: Cathodes	Full cells: Specific currents (A g^{-1})	Full cells: Cycles/Average capacity decay (%)	Ref.
$LiPF_6$@ ZIF-8	Li^+	45 wt.% EC/DMC/EMC	0.05 (Li)	500	$LiCoO_2$	0.05	100/0.1190	3
$LiPF_6$@PAF-1	Li^+	50 wt.% EC/DMC	NA (Li)	NA	$LiFePO_4$	0.68	1000/NA	4
3D-UIO-66/PAN/PEO/ LiTFSI	Li^+	NA	0.3 (60 °C) (Li)	700	$LiFePO_4$	~0.34 (60 °C)	300/0.0467	5
UN-SLi	Li^+	33 wt.% EC/DEC	1 (Li)	600	$LiFePO_4$	0.75	3000/0.0066	10
L@K/C	Li^+	NA	0.5 (Li)	600	$LiFePO_4$	~0.0340	300/0.052	11
CuBTC-PSS	Li^+	0.3 mg cm^{-2} (1M LiTFSI-PC)	1 (Li)	600	$LiFePO_4$	~0.17	500/NA	12
COF-NUST 7-8	Li^+	NA	0.05 (Li) (100 °C)	375	$LiFePO_4$	~0.0085 (100 °C)	60/0.1160	13
LGZ	Li^+	19.4 wt.% PC	0.1 (Li)	500	$LiFePO_4$	0.17	500/~0	14
LiOOC-COF3	Li^+	10 μL $LiPF_6$, EC/DEC v/v=1:1)	0.05 (Li)	320	C_6O_6	0.05	500/0.02057	15
DLC	Li^+	NA	0.3 (Li)	450	$LiFePO_4$	~0.017 (45 °C)	80/0.09615	16
NaFNFSI/ PEO	Na^+	NA	0.1 (80 °C) (Na)	200	$NaCu_{1/9}Ni_{2/9}Fe_{1/3}Mn_{1/3}O_2$	0.12 (80 °C)	150/0.2000	17
NaPTAB-SGPE	Na^+	66 wt.% PC	0.05 (60 °C) (Na)	100	$Na_3V_2(PO_4)_3$	0.06 (60 °C)	500~0.0615	18
NaOOC-COF	Na^+	10 mL, 1.0 M $NaPF_6$/PC	0.05 (Na)	700	BQ	0.2	600/0.0213	6
PLM@LE	Na^+	90 μL ($NaClO_4$-PC with 5% FEC	0.6 (Na)	~300	$Na_{0.44}MnO_2$	0.1	160/0.0606	8
TPDBD-CNa-QSSE	Na^+	~9 % solvent	0.01/0.05 (Na)	1000/ 450	$Na_3V_2(PO_4)_3$	0.06 0.12	1000/0.0048 500/0.0097	This work

*Unless otherwise noted, tests were conducted at 25 °C.

Supplementary Note 1

It should be noted that the interaction energy between TPDBD-CNa (–11 Kcal mol^{-1})/ Na^{+}(–1 Kcal mol^{-1}) and PC were calculated in TPDBD-CNa-NaTFSI/PC (Supplementary Figure 26e), and the stronger interaction energy between PC and TPDBD-CNa framework promotes the adsorption of PC solvents at the sub-nanometer zones, and a very small amount of PC may form solvated cations, and it was more difficult for the solvated PC to be removed from TPDBD-CNa-NaTFSI and then undergo oxidation.

Supplementary Note 2

The equivalent circuits of Ti|QSSEs|Ti symmetric cells were shown in Supplementary Figure 28-29. Two resistances (R) + constant phase elements (CPE) were used in series to model such phenomena. R_1 represents bulk resistance, R_2 corresponds to grain boundary resistance. CPE element describes the non-ideal capacitor behavior in solid-state conductors. CPE_3 is the electrode polarization[20].

Supplementary Note 3

The equivalent circuits of Na|QSSEs|Na symmetric cells were shown in Supplementary Figure 30. R_0 represents the bulk resistance, reflecting the resistance of electrodes, electrolyte, and separator. The semic-circle of R_1 in the high-middle frequency range represents the resistance of Na^+ transport through the SEI, while the semi-circle of R_2 in the low frequency range represents the de-solvation resistance of Na^+ before it enters the SEI[21].

Supplementary Note 4

$i_p = 2.69 \times 10^5 n^{3/2} A D_{Na}^{1/2} C_{Na} v^{1/2}$ $log(i) = log(a) + blog(v)$

CV curves of Na|TPBD-QSSE|NVP/C and Na|TPDBD-CNa-QSSE|NVP/C cells were showed in Supplementary Figure 43. The Na^+ diffusion coefficient (D_{Na^+}) can be calculated according to the following formula[22]

$$i_p = 2.69 \times 10^5 n^{3/2} A D_{Na}^{1/2} C_{Na} v^{1/2} \quad (1)$$

where i_p represents the peak current, stands for the surface area of the electrode, C_{Na} is the molar concentration of Na^+, n is the number of transferred electrons, and v is the sweep rate. Based on the fitted linear relationships between i_p and $v^{1/2}$, the D_{Na^+} value can be obtained.

Besides, the difference in kinetics between the peak 1 and peak 2 can be analyzed by a power law equation[23]

$$log(i) = log(a) + blog(v) \quad (2)$$

where i is the peak current, v is the scan rate, a and b are the fitting parameters. The b value reflects the reaction kinetics by estimating the ratio of capacitive-dominated charge storage of the electrode. The closer the b value is to 1, the more prominent the capacitive behavior is, which translates to faster reaction kinetics.

Supplementary Note 5

The cycling stability and rate performance of Na|TPDBD-CNa-QSSE|NVP/C cell with high cathode mass loadings of active materials (2.6 mg cm^{-2}) and thin Na anode (~100 μm) were carried out (Supplementary Figure 46). The Na|TPDBD-CNa-QSSE|NVP/C with high mass loading of NVP/C (2.6 mg cm^{-2}) retains 63 mAh g^{-1} after 100 cycles at 60 mA g^{-1} with a coulombic efficiency of 99.6%, and displays 99.7 mAh g^{-1} after 68 cycles at 12 mA g^{-1} with a coulombic efficiency of 99.6%. When the specific currents are 12, 24, 60, and 120 mA g^{-1}, the specific capacities of Na|TPDBD-CNa-QSSE|NVP/C are 101.5, 106, 91.6 and 78.5 mAh g^{-1}, respectively. Even at a higher rate of 240 mA g^{-1}, the cell still delivers 54.5 mAh g^{-1}. When the specific current returns to 60 mA g^{-1}, a specific capacity of 103.9 mAh g^{-1} can be obtained.

Supplementary References

1 Du, Y. *et al.* Ionic covalent organic frameworks with spiroborate linkage. *Angew. Chem. Int. Ed. Engl.* **55**, 1737-1741 (2016).

2 Park, S. S., Tulchinsky, Y. & Dincă, M. Single-ion Li^+, Na^+, and Mg^{2+} solid electrolytes supported by a mesoporous anionic Cu–azolate metal–organic framework. *J. Am. Chem. Soc.* **139**, 13260-13263 (2017).

3 Sun, C. *et al.* ZIF-8-based quasi-solid-state electrolyte for lithium batteries. *ACS Appl. Mater. Inter.* **11**, 46671-46677 (2019).

4 Zou, J., Trewin, A., Ben, T. & Qiu, S. High uptake and fast transportation of $LiPF_6$ in a porous aromatic framework for solid-state Li-ion batteries. *Angew. Chem. Int. Ed. Engl.* **59**, 769-774 (2020).

5 Li, Z. *et al.* A 3D interconnected metal-organic framework-derived solid-state electrolyte for dendrite-free lithium metal battery. *Energy Stor. Mater.* **47**, 262-270 (2022).

6 Zhao, G. *et al.* COFs-based electrolyte accelerates the Na^+ diffusion and restrains dendrite growth in quasi-solid-state organic batteries. *Nano Energy* **92**, 106756 (2022).

7 Zettl, R. *et al.* High Li^+ and Na^+ conductivity in new hybrid solid electrolytes based on the porous MIL-121 metal organic framework. *Adv. Energy Mater.* **11**, 2003542 (2021).

8 Zhang, G. *et al.* Pancake-like MOF solid-state electrolytes with fast ion migration for high-performance sodium battery. *Nano-Micro Lett.* **13**, 105 (2021).

9 Fischer, S. *et al.* A metal–organic framework with tetrahedral aluminate sites as a single-ion Li^+ solid electrolyte. *Angew. Chem. Int. Ed.* **57**, 16683-16687 (2018).

10 Shi, W. *et al.* Electrolyte membranes with biomimetic lithium-ion channels. *Nano Lett.* **20**,

5435-5442 (2020).

11 Sun, W. *et al.* Ultrathin aramid/COF heterolayered membrane for solid-state Li-metal batteries. *Nano Lett.* **20**, 8120-8126 (2020).

12 Chang, Z., Yang, H., Zhu, X., He, P. & Zhou, H. A stable quasi-solid electrolyte improves the safe operation of highly efficient lithium-metal pouch cells in harsh environments. *Nat. Commun.* **13**, 1510 (2022).

13 Shan, Z. *et al.* Covalent organic framework-based electrolytes for fast Li^+ conduction and high-temperature solid-state lithium-ion batteries. *Chem. Mater.* **33**, 5058-5066 (2021).

14 Jiang, G. *et al.* Glassy metal–organic-framework-based quasi-solid-state electrolyte for high-performance lithium-metal batteries. *Adv. Funct. Mater.* **31**, 2104300 (2021).

15 Zhao, G. *et al.* COF-based single Li^+ solid electrolyte accelerates the ion diffusion and restrains dendrite growth in quasi-solid-state organic batteries. *Carbon Energy*, 1-13 (2022).

16 Guo, D. *et al.* Foldable solid-state batteries enabled by electrolyte mediation in covalent organic frameworks. *Adv. Mater.* **34**, e2201410 (2022).

17 Ma, Q. *et al.* A new $Na[(FSO_2)(n\text{-}C_4F_9SO_2)N]$-based polymer electrolyte for solid-state sodium batteries. *J. Mater. Chem. A* **5**, 7738-7743 (2017).

18 Yang, L. *et al.* Novel sodium–poly(tartaric acid)borate-based single-ion conducting polymer electrolyte for sodium–metal batteries. *ACS Appl. Energy Mater.* **3**, 10053-10060 (2020).

19 Ashraf, S. *et al.* Versatile platform of ion conducting 2D anionic germanate covalent organic frameworks with potential for capturing toxic acidic gases. *ACS Appl. Mater. Inter.* **12**, 40372-40380 (2020).

20 Li, J. *et al.* Room temperature all-solid-state lithium batteries based on a soluble organic cage

ionic conductor. *Nat. Commun.* **13**, 2031 (2022).

21 Wu, J. *et al.* A synergistic exploitation to produce high-voltage quasi-solid-state lithium metal batteries. *Nat. Commun.* **12**, 5746 (2021).

22 Zhang, J. *et al.* Understanding the superior sodium-ion storage in a novel $Na_{3.5}Mn_{0.5}V_{1.5}(PO_4)_3$ cathode. *Energy Stor. Mater.* **23**, 25-34 (2019).

23 Rui, X., Sun, W., Wu, C., Yu, Y. & Yan, Q. An advanced sodium-ion battery composed of carbon coated $Na_3V_2(PO_4)_3$ in a porous graphene network. *Adv. Mater.* **27**, 6670-6676 (2015).
